# SNSMIL, a real-time single molecule identification and localization algorithm for super-resolution fluorescence microscopy

**DOI:** 10.1038/srep11073

**Published:** 2015-06-22

**Authors:** Yunqing Tang, Luru Dai, Xiaoming Zhang, Junbai Li, Johnny Hendriks, Xiaoming Fan, Nadine Gruteser, Annika Meisenberg, Arnd Baumann, Alexandros Katranidis, Thomas Gensch

**Affiliations:** 1 National Center for Nanoscience and Technology of China No.11, Beiyitiao Zhongguancun 100190 Beijing, P.R. China; 2 Department of Physics, Chongqing University, Chongqing 400044, P.R. China; 3 State Key Laboratory of Theoretical Physics, Institute of Theoretical Physics, Chinese Academy of Sciences, 100190 Beijing, P.R. China; 4 Institute of Chemistry, Chinese Academy of Sciences, 100190 Beijing, P.R. China; 5 Institute of Complex Systems (ICS-4, Cellular Biophysics), Forschungszentrum Jülich, Leo-Brandt-Str., 52428 Jülich, Germany; 6 Institute of Complex Systems (ICS-5, Molecular Biophysics), Forschungszentrum Jülich, Leo-Brandt-Str., 52428 Jülich, Germany

## Abstract

Single molecule localization based super-resolution fluorescence microscopy offers significantly higher spatial resolution than predicted by Abbe’s resolution limit for far field optical microscopy. Such super-resolution images are reconstructed from wide-field or total internal reflection single molecule fluorescence recordings. Discrimination between emission of single fluorescent molecules and background noise fluctuations remains a great challenge in current data analysis. Here we present a real-time, and robust single molecule identification and localization algorithm, SNSMIL (Shot Noise based Single Molecule Identification and Localization). This algorithm is based on the intrinsic nature of noise, *i.e.*, its Poisson or shot noise characteristics and a new identification criterion, *Q*_SNSMIL_, is defined. SNSMIL improves the identification accuracy of single fluorescent molecules in experimental or simulated datasets with high and inhomogeneous background. The implementation of SNSMIL relies on a graphics processing unit (GPU), making real-time analysis feasible as shown for real experimental and simulated datasets.

The past century has seen an ever intensifying demand of science and technology to investigate structure, composition and state of matter on increasingly smaller length scales. Consequently, this led to an unforeseen and impressive role for microscopy techniques that exist now in many modalities exploiting a great variety of physical and chemical processes (*e.g.*, neutron or x-ray scattering and diffraction, atomic force and conductance measurements, infrared absorption). Since its invention a hundred years ago (Otto Heimstädt and Heinrich Lehmann, between 1911 and 1913) fluorescence microscopy^1^,^2^ has been developed enormously to the most versatile non-invasive microscopy modality of today. Its primary application fields are material nanoscience and modern cell biology.

There exists, however, a severe limitation in the performance of an optical (here fluorescence) microscope – that is its non-infinitely small resolution capacity as has been formulated already in the late 19th century^3^,^4^. Using the best modern high numerical aperture (NA) oil-immersion microscope objectives, a maximal lateral resolution between 160 nm and 250 nm can be achieved (for detection wavelengths from 400 nm to 750 nm). This causes a great limitation for the use of fluorescence microscopy, since many cellular structures (*e.g.*, mitochondria, endoplasmic reticulum, Golgi apparatus) as well as morphological features in nanostructured materials and devices have dimensions in the range from 5 to 500 nm.

This limitation of fluorescence microscopy was an accepted fact by the research community for more than 100 years. Since about 20 years, however, theoretical schemes and practical realizations of fluorescence microscopy have been developed that allow imaging with a resolution better than the diffraction limit. The techniques can be grouped into two classes. In one class the illumination conditions of the sample are changed in a way that allow a resolution enhancement, *e.g.*, a smaller effective point spread function (PSF) (stimulated emission depletion, STED^5^) or spatial modulations of the excitation light distribution (structured illumination microscopy, SIM^6^,^7^). The other class – subsumed as single molecule localization microscopy (SMLM; *e.g.*, PALM^8^, FPALM^9^, STORM^10^, dSTORM^11^, GSDIM^12^, SPDM^13^) is based on the detection of the fluorophores as single emitters and the determination of their positions. The crucial point of SMLM is the use of fluorophores that can populate a non-fluorescent state and the possibility to switch between non-fluorescent and fluorescent states. Biological applications of SMLM grow rapidly and range from bacteria^14^, cell biology^15^,^16^,^17^ to neuroscience^18^,^19^ and immunology^20^.

A key aspect of SMLM experiments is the determination of the single emitter positions. The development of SMLM, however, occurred in several laboratories in parallel. Therefore, there is no standard analysis program-package available for the SMLM community but a multitude of them. There are many stand-alone programs available (*e.g.*, DAOSTORM^21^, livePALM^22^, rapidSTORM^23^, MLEwT^24^, Localizer^25^, deconSTORM^26^, 3D-DAOSTORM^27^, WaveTracer^28^, GPUgaussMLE^29^) as well as plugins for the free graphics program ImageJ^30^ (*e.g.*, QuickPALM^31^, MaLiang^32^, GrasPJ^33^). Some programs use GPU to accelerate analysis (*e.g.*, GPUgaussMLE, WaveTracer, MaLiang). Many laboratories use their own localization software or the programs delivered with the commercial SMLM microscopes (*e.g.*, Elyra (Zeiss), N-STORM (Nikon), SR GSD (Leica)). Two recent publications give an excellent overview about the different problems and approaches for localization of single molecules^34^,^35^.

All SMLM program packages require that the user needs to choose a set of parameters that influence the performance of the program and have a considerable impact on the super-resolution image obtained. It is, however, rather difficult for experimentalists not involved in the development of the analysis programs or not trained in single molecule microscopy to perceive and predict the influence of a certain parameter on the final result (the super-resolution image) and it is therefore complicated to make reasonable choices. Such a situation is nowadays occurring with increasing probability due to the growing popularity of SMLM methods among cell biologists, physiologists, neuroscientists or microbiologists – inspired by the Nobel Prize in Chemistry 2014 awarded partly to the basics of SMLM. Non-experts in single molecule imaging start using SMLM as an established imaging tool as can be seen from the growing number of publications that use rather than develop or improve SMLM and the spread of commercial SMLM setups. Our intention was to develop a simple-to-use algorithm with very few adjustable parameters and prove its suitability for simulated and real SMLM data from ideal (simple) to complicated imaging conditions. SNSMIL allows the generation of super-resolution images from SMLM data where the acceptance of the single molecule localizations is decided purely on the basis of the quality of the detected single molecule signals.

Due to experimental implementation and sample preparation, background is uneven in many measurements, therefore, local SNR (Signal-to-Noise Ratio) analysis is essential and being used in excellent SMLM software such as rapidSTORM, QuickPALM, Localizer, MaLiang, *etc*.. For a discussion of different SNR definitions we refer to a recent publication^36^. SNR analysis depends on the local background estimation. Hoogendoorn *et al.*^37^ recently suggested an interesting different approach to correct for the contribution of background signal in SMLM. They applied a temporal median filter to estimate the background in SMLM measurements and demonstrated its usefulness in generating super-resolution images from SMLM datasets. Here, we introduce a new single molecule localization program-package (SNSMIL, Shot Noise based Single Molecule Identification and Localization, source code and binary program available online ( http://english.nanoctr.cas.cn/dai/software/)). The algorithm of SNSMIL is based on the principle of noise source, namely shot noise^38^ or Poisson noise of an image acquired with an EMCCD camera. SNSMIL applies the Rose criterion^39^,^40^,^41^,^42^,^43^,^44^ and introduces a newly defined quality metric, 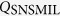. This algorithm allows users to generate a super-resolution image by choosing only one parameter - the threshold of 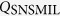
- if the width-fixed Gaussian fit model is used. In some cases, especially when the imaged biological structures extend in z-direction, a variable width in the Gaussian fitting model is needed to allow “out of focus” single molecules to be accepted. For those cases a second parameter - localization precision filtration for single emitters – is introduced. All other settings in SNSMIL are dictated by either the used equipment or the used fluorophores. As such we are able to obtain reliable and comparable results by making use of the single emitter quality metric 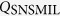, even when different acquisition modes (*e.g.* PALM or dSTORM) are used. We describe here the principle and the implementation of SNSMIL as well as its application on simulated and measured PALM and dSTORM data. The performance of SNSMIL is compared to several other SMLM program packages.

## Results

To generate a SMLM-based super-resolution image, three consecutive procedures are performed in general, step 1: acquisition of a series of fluorescence images (frames) of single emitters; step 2: identification of potential single molecule fluorescence emitters in each frame; step 3: localization of the central position of each emitter and reconstruction of the final image. The single molecule identification strategy (step 2) is the central problem for all SMLM algorithms^21^,^22^,^23^,^24^,^25^,^26^,^27^,^28^,^29^,^30^,^31^,^32^,^33^. SNSMIL defines a new metric for identification, 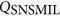, that allows for an easy assessment of the quality of an identified single molecule. In the following paragraphs a detailed description of the different elements and formulas that are involved in this step are given.

SNSMIL makes use of shot noise to evaluate if a region in an image contains a potential single molecule fluorescence emitter. The use of shot noise requires that the intensity reading of each pixel is first converted into the number of photoelectrons. This conversion is done using equation (1), where 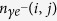 and *I*(*i*, *j*) denote the number of photoelectrons and intensity reading of pixel (*i*,*j*); 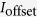 denotes the bias offset of the camera; and *G*_eff_ indicates the applied effective electronic gain (see Supplementary Information Part 1, section 2.3.2 for details).


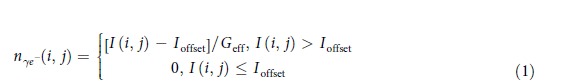


Due to the fundamental nature of shot noise, which is Poisson distributed, and the excess noise factor of the camera, the overall background noise in a pixel, 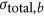, acquired on a camera with low sensor temperature and high electronic gain can be formulated as equation (2)^45^,^46^,^47^. Here 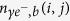 denotes the number of background photoelectrons of pixel (*i*,*j*) and *F* denotes the excess noise factor of the camera, which equals 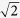 for EMCCD cameras^47^.


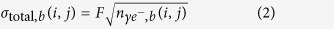


The signal-to-noise ratio (SNR) for pixel (*i*,*j*) can now be determined using equation (3).


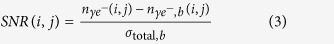


To find potential single molecule fluorescence emitters within an image, each pixel of the image is evaluated to determine whether it contains the maximum intensity value within the airy radius (*R*_airy_) defined by equation (4). Here *M* is the optical magnification; *λ* is the wavelength of maximum fluorescence emission; *μ* is the pixel size of the camera; and NA is the numerical aperture of the imaging system.


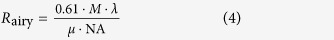


If a pixel contains the maximum value within the airy radius it is considered as an emitter candidate(*i*_*peak*_, *j*_*peak*_). Each candidate is then re-evaluated by defining a region of interest (ROI) around the candidate that encompasses all pixels within the theoretical width of the PSF (eq. 5)


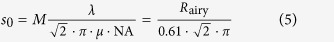


which meet the condition in equation 6


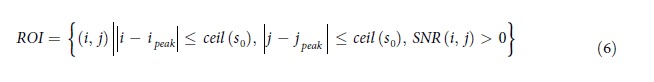


where ceil() rounds the elements to the nearest greater or equal integer number. The purpose of this ROI selection is to exclusively include pixels into the analysis that have a signal larger than the background. For each ROI the effective signal-to-noise and contrast to noise ratio (*SNR*_eff_and*CNR*_eff_) is calculated using equations (7) and (8), where 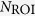 denotes the number of pixels in the ROI.


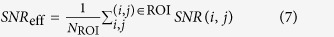



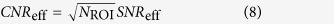


In order for a pixel to be considered the position of a potential single molecule fluorescence emitter, it must pass two constraints. According to the Rose Criterion, a signal is detectable if *CNR*_eff_ is larger than three^39^,^40^,^41^,^42^,^43^,^44^. This is the first constraint and cannot be influenced by the user. The second constraint is the identification quality metric 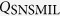 defined by equation (9).


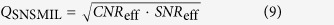


The threshold of 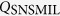 is the only parameter to be chosen by the user that determines the qualification of potential emitters. It represents the central idea of SNSMIL. Spike-like single pixel signals (needle-like) have a high SNR but a low CNR. Broadened signals (with a width significantly larger than the theoretical PSF width) typically have a high CNR and a low SNR. Both situations are unlikely to be caused by a single emitter. 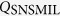 accounts for both situations and when set correctly will remove false positives caused by either spike-like or broad signals. In practice, several values should be empirically tested to obtain the optimal result for each recording.

The identified potential single molecule fluorescence emitters are then analyzed in step 3, *i.e.*, the localization of the central position of the emitters and the subsequent reconstruction of the super-resolution image. SNSMIL tries to fit a two-dimensional Gaussian function using the Levenberg-Marquardt algorithm (LMA)^48^,^49^, to determine the central position of each potential emitter. SNSMIL makes use of a GPU implementation of the LMA to solve this non-linear curve fitting problem. The 2D Gaussian model can be used in three different variations in SNSMIL (model 1–3; see Supplementary Information Part 1, Appendix A for details). In model 1 the width in both the x and y direction is fixed to the theoretical PSF width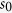. In model 2 the width is a fit parameter that is the same for both the x and y direction. Both models 1 and 2 result in a symmetrical 2D Gaussian function. In model 3 the width for the x and y direction are fit independently of each other, which results in an elliptical 2D Gaussian function. In case an emitter is slightly out of focus or if the background is high, the PSF broadens and the localization accuracy is decreased. Therefore, models 2 and 3 make use of the PSF-width tolerance (*PWT*) parameter to limit the valid range for the width parameter values that are obtained in the fit. The minimum allowable width is essentially based on the pixel-size of the image. The maximum allowable width is equal to 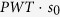. Potential single molecule fluorescence emitters with widths outside the valid range will be discarded. See Supplementary Information Part 1, section 3.8 for details.

**Figure 1:**
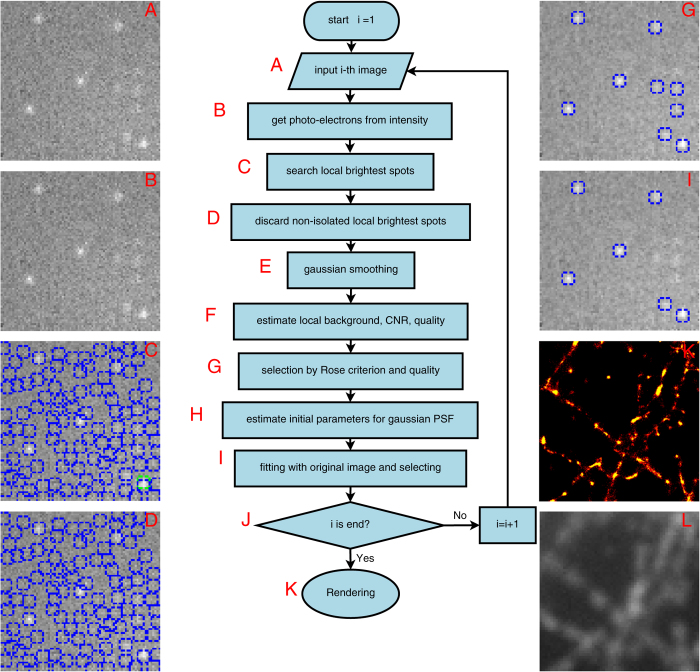
SNSMIL Work Flow. Schematic diagram of SNSMIL work flow (**A**–**J**), super-resolution image (**K**), and TIRF image (**L**) of tubulin fibers obtained from fixed HL-1 cells immunohistochemically stained with anti-β-tubulin primary and Alexa647 labeled secondary antibodies.

For a better general understanding of the algorithm, the work flow of SNSMIL is shown in Fig. 1. After an image has been acquired (step A), the image intensity is transformed into photoelectrons (step B), which allows shot noise analysis using a Poisson distribution. The locally brightest spots are chosen as candidates in step C. Subsequently non-isolated candidates of emitters (the distance between two emitters is less than the radius of the airy disc Rairy) are discarded in step D. Noise is then reduced via Gaussian smoothing in step E. In step F, CNR and 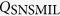
are estimated by shot noise analysis using a Poisson distribution. Candidates are further filtered in step G based on the Rose criterion (CNR > 3) and the user provided threshold 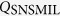. The initial value of parameters are estimated before fitting in step H. The fitting process is performed for all detected emitters in the image and a post-fitting filter is applied to identify emitters with desired goodness of fit defined by Pearson’s correlation coefficient in step I (see Supplementary Information Part 1, section 3.8.3 for details). The final super-resolution image is generated and rendered in the last step K.

SNSMIL was tested with three simulated datasets comprised of different signal and noise contributions, high SNR, low SNR and low SNR with GB (see *Image Simulations* in *Methods*). We used four indexes: Jaccard, Precision, Recall, and RMSD (root mean square distance) to evaluate the performance. These indexes are defined by equations (10)–(13). Here we denominate *A* as the simulated dataset (reference dataset) and *B* as the reconstructed dataset. Three relevant quantities are defined as true positive (*TP*), false positive (*FP*) and false negative (*FN*) as follows: the number of true emitters that are reconstructed, *TP* = *A* ∩ *B*; the number of false emitters that are reconstructed, 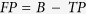; and the number of true emitters that are missed, 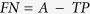.


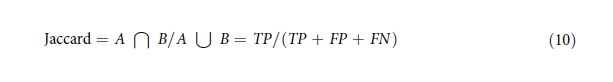



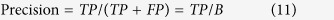



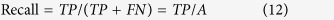



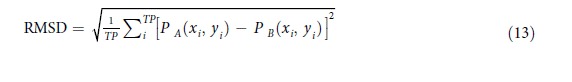


**Figure 2:**
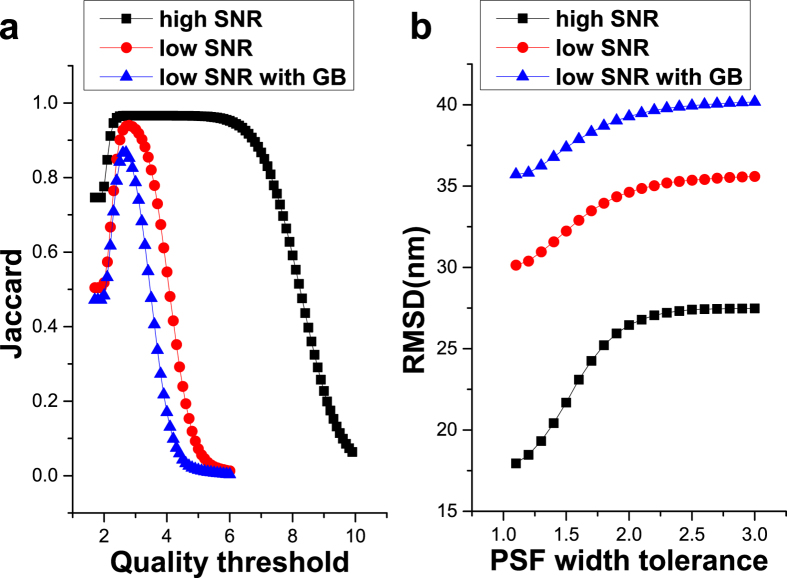
Identification and localization precision with three simulated datasets. (**a**) Constant precision parameters (PWT = 3.0) and variable identification parameter (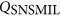) by using fixed width 2D Gaussian Model. (**b**) Constant identification parameter (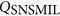 = 2.0) and variable precision parameters (PWT) by using symmetrical but variable width 2D Gaussian Model.

The Jaccard similarity coefficient is a measure for the similarity between simulated and reconstructed emitters and is the most important representative for identification accuracy. In Fig. 2a the influence of the 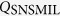 parameter on the Jaccard coefficient is shown. It is clear that choosing a value for 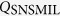, which is either too low or too high has a negative influence on the Jaccard coefficient. Under ideal imaging conditions, good results are insensitive for a large range of 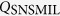 values since the difference between signal and background is significant. In contrast, under less ideal imaging conditions that are closer to experimental reality, the 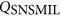 value needs to be selected carefully.

The influence of *PWT* on identification sensitivity and localization precision is shown in Fig. 2b. A higher *PWT* setting results in a worse localization precision (higher RMSD), however in certain cases, *e.g.*, when dealing with a large set of emitters (high sampling density), the tradeoff of a higher RMSD may be beneficial for resolving fine structure.

**Figure 3:**
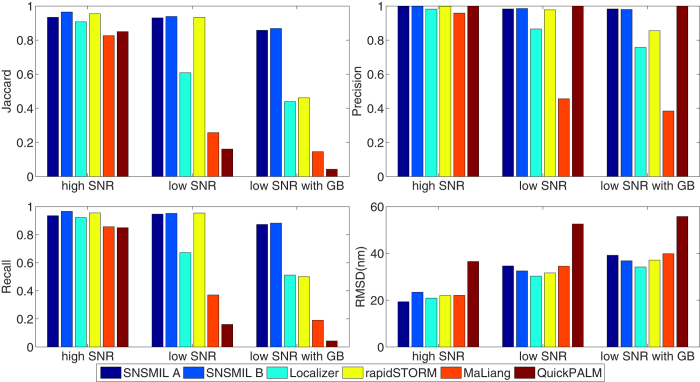
Performance comparison of SMLM software. Performance of software with three simulated datasets. SNSMIL A uses a symmetrical but variable width 2D Gaussian Model with 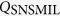 = 2; SNSMIL B, uses a fixed width 2D Gaussian Model with 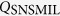 = 2. The settings and results of parameters and retrieved super-resolution images for all software can be found in the Supplementary Information Part 2 (Fig. S1–S22).

The performance of SNSMIL is compared with some of its precursors, QuickPALM 1.1, rapidSTORM 3.2, Localizer 88, and MaLiang 1.1 in Fig. 3. We tested a number of parameter sets for each program and performed the analysis with each program with the best possible found parameter set. Performances with different parameter settings of rapidSTORM in the case of Low SNR with GB are shown as an example in Fig. S22. The detailed parameter settings and retrieved super-resolution images are depicted in Supplementary Information Part 2 (Fig. S1–S22). SNSMIL performs well and similar to rapidSTORM and Localizer while the results of MaLiang and QuickPALM are of lower quality. SNSMIL with both width-variable (SNSMIL A) and width-fixed (SNSMIL B) 2D Gaussian fitting model were tested. SNSMIL B gives better Jaccard and RMSD in worse imaging conditions (Low SNR and Low SNR with GB). It is suggested for applications when emitters are mostly in focus, for instance in the case of TIRF illumination. However, SNSMIL A accepts emitters when the width of the fitted Gaussian functions is distributed in a certain range and is recommended for imaging conditions, where emitters are located in but also slightly out of focus, for instance in the case of EPI illumination. The times used for computation by different software packages are listed in Table 1. SNSMIL processes single frames in a parallel fashion, that is to say, emitters identified in one frame are processed at the same time in different computing threads so that the localization results are achieved only with a short time lag after the frame have been recorded. Both rapidSTORM and MaLiang use a different strategy of parallel computing, *i.e.*, they process several frames at the same time. In this way, they are considerably faster in computation of the simulated datasets compared to SNSMIL. However, the conceptual advantage of paralleled processing of only one single frame consists in its perfect match for real-time analysis and reconstruction of SMLM measurements, where only one image at a time is available for computation. As shown below, SNSMIL performance speed is adequate to process SMLM data in real-time in measurements of mitochondrial structures in HEK293 cells.

**Figure 4:**
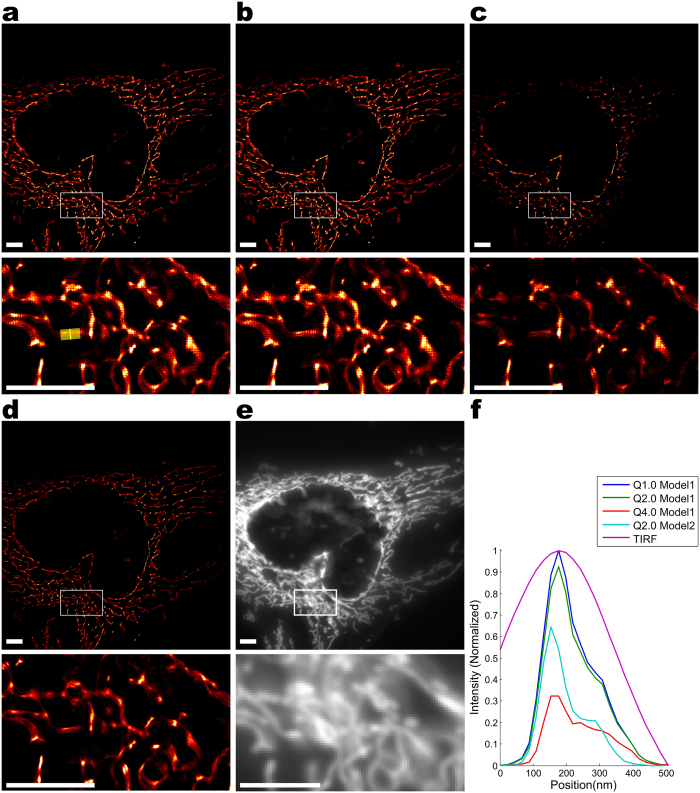
PALM imaging. PALM imaging of mitochondria (mito-Dendra2 in HEK293 cells) analyzed by SNSMIL: In 4a–4d the super-resolution images generated with SNSMIL under different parameter settings are shown. In 4e the corresponding TIRF image is depicted. An enlarged view of the boxed region is shown below each image. Fitting model, 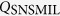 value, and number of emitters reconstructed for the whole and the zoomed images are listed below: (**a**) Model 1; 2.0; 3,270,739; 245,259 (**b**) Model 1; 1.0; 3,785,947; 262,522 (**c**) Model 1; 4.0; 947,779; 104,470 (**d**) Model 2; 2.0; 1,825,069; 132,350 (PWT is fixed as 3.0). A selected finger-like structure is marked by a yellow box (4a, lower part) and projected onto the x-axis marked as yellow line. (**f**) Line profiles of a straight mitochondrial structure were plotted for images 4a–4e. Each line profile through a reconstructed SMLM image is normalized to its maximal intensity. Intensities from the line profile through the same structure in the corresponding TIRF image are normalized to match the same scale. Scale bar is 4 μm.

**Figure 5:**
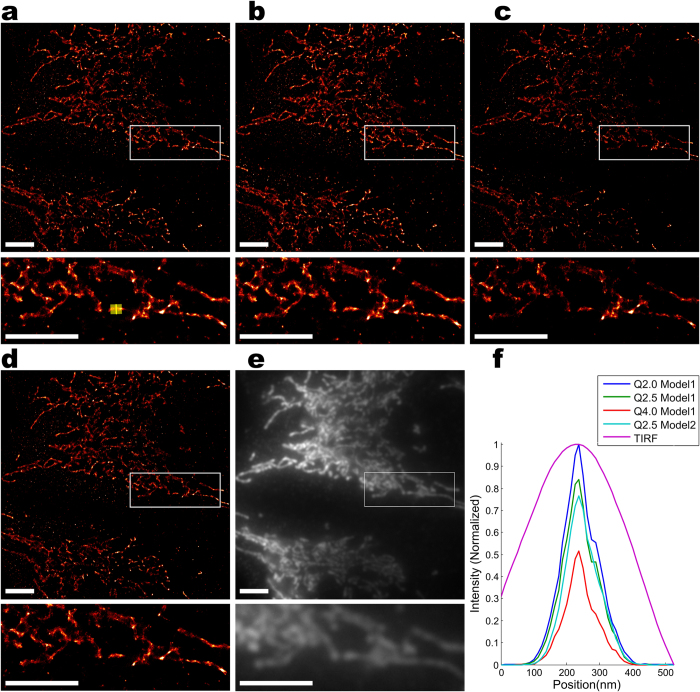
dSTORM imaging. dSTORM imaging of mitochondria (immunohistochemical staining of mito-GCaMP3 (secondary antibody labeled with Alexa647)) in HEK293 cells analyzed by SNSMIL: In 5a–5d super-resolution images generated under different parameter settings are shown. In 5e the corresponding TIRF image is depicted, where the contrast of the lower image is re-adjusted for better visibility. An enlarged view of the boxed region is shown below each image. Fitting model, 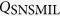 value, and number of emitters reconstructed for the whole and the zoomed images are listed below: (**a**) Model 1; 2.5; 1,362,080; 124,916; (**b**) Model 1; 2.0; 1,648,679; 147,744; (**c**) Model1; 4.0; 792,544; 74,727 (**d**) Model 2; 2.5; 1,197,259; 102,409 (PWT is fixed as 3.0). A selected finger-like structure is marked by a yellow box (5a, lower part) and projected onto the x-axis marked as yellow line. (**f**) Line profiles of a straight mitochondrial structure were plotted for images 5**a**–5**e**. Each line profile through a reconstructed SMLM image is normalized to its maximal intensity. Intensities from the line profile through the same structure in the corresponding TIRF image are normalized to match the same scale. Scale bar is 4 μm.

SNSMIL was also tested on real SMLM measurement data. We analyzed images of mitochondria in HEK293 cells obtained from photoactivated localization microscopy (PALM; experiment A; Fig. 4) and direct stochastic optical reconstruction microscopy (dSTORM; experiment B; Fig. 5). In both cases the fluorophore (Dendra2 in experiment A or Alexa647 in experiment B) is directed to the mitochondrial matrix due to the same targeting sequence (see *Protein constructs* in *Methods*). For PALM experiments, Dendra2 was directly fused to the mitochondrial matrix targeting sequence and in this way transported into the mitochondrial matrix. dSTORM experiments were performed with a HEK293 cell line stably expressing mito-GCaMP3, a genetically encoded GFP-based Ca^2+^ sensor. Visualization occurred via immunocytochemistry using a primary antibody against GFP and a secondary antibody labeled with Alexa647. In both experiments, the mitochondria were intensely stained while hardly any fluorescence was detected outside the mitochondria. The resolution of the SMLM images were explicitly and significantly improved compared to the conventional total internal reflection fluorescence (TIRF) images (compare Fig. 4a/5a with Fig. 4e/5e).

To meet real-time analysis, SNSMIL is implemented on GPU to make use of GPU’s parallel computation power. With typical parameters, analysis with SNSMIL for experiment A and B are finished in 133 s (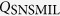 = 2; *PW* = 3; 3914 frames; 836 emitters and 34 ms per frame on average) and 119 s (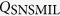 = 2.3; *PWT* = 3; 4000 frames; 340 emitters and 30 ms per frame on average), while the measurements took 391 seconds (about 100 ms per frame) and 224 seconds (about 56 ms per frame), respectively. This shows that real-time analysis can be achieved when SNSMIL is embedded into a measurement program.

For both, experiment A and B, a thin finger-like mitochondrial structure is selected for resolution analysis. Line profiles of these structures in SMLM as well as conventional TIRF images were obtained by projecting the localizations on a perpendicular axis shown in Figs 4f and 5f. In the TIRF images, the widths of the mitochondria are 200–300 nm corresponding to the diffraction limited resolution. The significantly smaller widths in the SMLM images amount to only 80–120 nm. Additional fine structures, which were unresolved in the TIRF images, can also be seen in the SMLM images (see Figs 4 and 5).

The effects of different 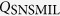 settings and different fitting models were also investigated (Fig. 4a–d and Fig. 5a–d). Model 1 is the model of choice for SMLM data that are analyzed as twoαdimensional projections (as is the case here), models 2 and 3 are favorable for several of the three-dimensional SMLM methods like biplane^50^ or cylindrical lens^51^. With the same 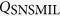, different fitting models result in different sets of recovered emitters due to localization precision filtering. Interestingly, fine structures are more evident with a moderate amount of emitters reconstructed. With other parameter sets, fine structures may become blurry with too large or too small emitter sets corresponding to low precision or low recall as visible in Fig. 4f.

**Figure 6:**
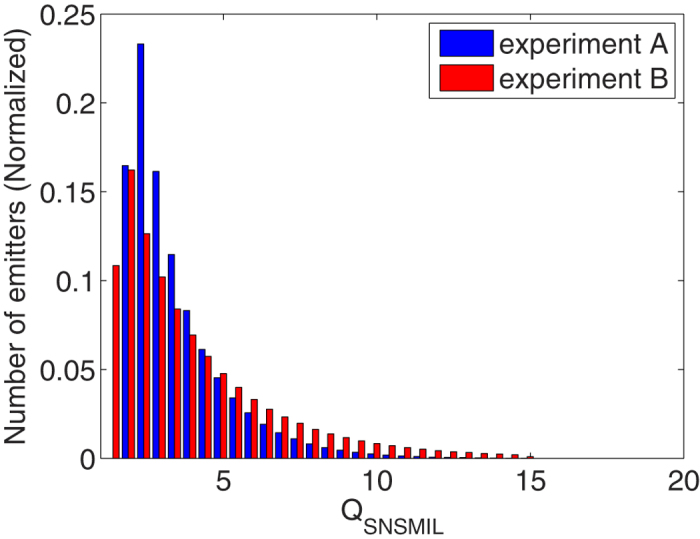
QSNSMIL histograms. Distributions of the quality 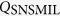 of the PALM (experiment A; Fig. 4) and dSTORM (experiment B; Fig. 5) super-resolution data.

The histograms of 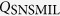 for the two experiments, the quality distribution of emitters is shown in Fig. 6. The comparison clearly shows that there are more emitters with high 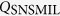 in experiment B compared to experiment A, and the mean values of 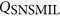 in experiment A and B are 3.39 and 4.27, respectively. This finding is consistent with the fact that Alexa647 is a brighter fluorophore than Dendra2 and therefore better to distinguish from background. The distribution of the 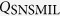 values of SMLM data offers a valuable method to characterize the quality of a SMLM measurement and to choose an appropriate 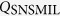
for generating a reasonable super-resolution image.

## Conclusions

We have developed SNSMIL, a real-time and robust single molecule identification and localization algorithm and software for SMLM. SNSMIL is implemented to make use of a GPU to reduce computation time and make real-time analysis possible. We demonstrate SNSMIL as a stand-alone program (BSD license) with a graphical user interface, that can be executed on Windows or Linux systems installed with NVidia CUDA enabled graphics cards (with compute capability 2.0 or higher). We demonstrate that SNSMIL provides robust and high identification accuracy even when imaging quality is rather limited, namely high background or high noise conditions. This holds true for both, simulated – where SNSMIL performs well compared to other established SMLM fitting programs – as well as in real super-resolution experiments uncovering details of cellular structures well below the diffraction limited resolution of optical microscopy.

## Methods

### Image Simulation

To evaluate the performance, three simulation datasets were produced with different imaging conditions: high signal-to-noise ratio (high SNR, average number of photons from an emitter is 500 with an uniform background of 10 photons per pixel), low signal-to-noise ratio (low SNR, average number of photons from an emitter is 500 with an uniform background of 50 photons per pixel), and low signal-to-noise ratio with inhomogeneous Gaussian distributed background (low SNR with GB, average number of photons from an emitter is 500 with an uniform background of 50 photons per pixel, where an additional Gaussian distributed background is applied with a width of half size of full image, and the peak value of the Gaussian distributed background is 50 photons). In the last case, a broad Gaussian distribution was superimposed on each frame, which is often the case in wide-field and TIRF illumination. Photons from emitters were randomly placed within a PSF area, and then shot noise was imposed after adding a constant offset to simulate a photoelectron image. The image is further multiplied with an EMCCD gain factor to obtain the intensity image. The PSF is simulated with the following parameters: pixel size 64 nm; emission wavelength 665 nm and numerical aperture 1.49. Each dataset contains 200,000 emitters in about 5,000 frames to produce robust statistic comparable results. The simulation datasets are generated with a program coded in Matlab. The comparisons between reference and reconstructed datasets are performed by the evaluation tool (CompareLocalization)^52^ released by Localization Microscopy Challenge.

### Computation system architecture

Computation with SNSMIL were performed on a GPU based computer (GPU: Nvidia GTX 580; CPU intel core i5–3470(3.2 GHz); 4 GB memory; Operating system is Debian Linux 6.0 64 bit).

### Protein constructs

A pMC vector for mammalian expression containing a YFP construct that is targeted to the mitochondrial matrix was kindly provided by Dr. M. O. Christensen (Heinrich Heine University Düsseldorf, Düsseldorf, Germany). A fragment encoding the amino-terminal 29 amino acids of the precursor of subunit VIII of cytochrome c oxidase (Mitochondrial Targeting Sequence – MTS)^53^ had been fused 5’-upstream of the YFP gene. The YFP-encoding fragment was excised from the pMC vector with AgeI and SalI, leaving the MTS unaffected. The gene encoding Dendra2 (Addgene, Cambridge, MA, USA) was amplified from a pET vector by PCR. Unique recognition sites for AgeI and SalI were added at the N- and C-terminal end, respectively. The PCR fragment was ligated into the pMC vector downstream the MTS between the AgeI and SalI restriction sites yielding an expression plasmid for a photoconvertible fluorescent protein targeted to the mitochondrial matrix (mito-Dendra2). The construct was verified by DNA sequencing. The GFP-based Ca^2+^ sensor GCaMP3^54^ was fused in a similar manner to the same targeting sequence for the mitochondrial matrix yielding mito-GCaMP3^55^.

### Cell Culture

HEK293 cells – either stably transfected with mito-GCaMP3 or transiently transfected with mito-Dendra2 by a modified Ca^2+^-phosphate method^56^ were maintained in minimum essential medium (MEM) supplemented with 10% (v/v) fetal calf serum (FCS), 1% (v/v) streptomycin/penicillin and 1% (v/v) essential amino acids (Life Technologies, Darmstadt, Germany) at 37 °C in a humidified, CO_2_-controlled (5%) incubator. 24 h before dSTORM experiments 2 × 10^4^ cells were plated on 8-well μ-Slides (ibiTreat, ibidi, Martinsried, Germany) coated with 150 μl poly-L Lysine (0.1 mg/ml). Coating was performed for 30 min at RT before cells were plated. Wells were rinsed three times with sterile filtered PBS.

### Immunocytochemical Staining

To reduce background fluorescence all solutions were sterile filtered. All incubation steps were performed at room temperature (RT). Cells were washed three times with PBS and subsequently incubated in 4% paraformaldehyde (PFA) in PBS for 15 minutes. After fixation the cells were rinsed six times for 5 minutes in PBS and permeabilized for 10 minutes at RT in 250 μl PBS containing 0.5% (v/v) Triton X-100. Triton X-100 was removed by washing three times (5 minutes each) with PBS. Afterwards cells were incubated in 150 μl blocking buffer containing 5% (v/v) normal goat serum in sterile filtered PBS for 45 minutes at RT. Subsequently cells were incubated in 150 μl blocking buffer containing primary antibodies against GFP (mouse anti-GFP (MAB3580), Chemicon (Millipore, Schwalbach, Germany, dilution 1:8000)) for 60 minutes at RT. After washing the samples six times with 0.1% (v/v) Tween-20 in PBS for 5 minutes each they were incubated for 60 minutes in the dark with secondary antibodies conjugated with Alexa647 (Alexa Fluor 647 F(ab’)2 fragment goat anti-mouse IgG (A-21237), Life Technologies, dilution 1:10000) diluted in blocking buffer. Finally, all samples were washed six times with 0.1% (v/v) Tween-20 in PBS for 5 minutes each. Samples were again fixed with 4% PFA solution for 5 minutes to preserve the staining. For labeling of tubulin fibers (Fig. 1K,L) mouse anti-β tubulin primary antibodies (32–2600; Life Technologies; dilution 1:500) were used in conjunction with the same secondary antibody as mentioned above.

### Wide-field/TIRF fluorescence microscope

Our custom built setup is based on an Olympus IX-71 inverted microscope body (Olympus, Hamburg, Germany). We use an AOTF (AOTF nC-VIS-TN 1001; AA Opto-Electronic, Orsay, France) to control the throughput of three continuous wave laser sources, *i.e.*, an Ar-ion laser (488 nm; Innova 70C; Coherent, Santa Clara, CA, USA), a 561 nm diode laser (Sapphire 561-200 CDRH-CP; Coherent), and a 642 nm diode laser (LBX-642-130 CIR-PP; Oxxius, Lannion, France). In addition, we use a 405 nm diode laser (Cube 405-100C; Coherent), which is controlled via a digital/TTL signal. All measurements were done with an ApoN 60x Oil TIRF objective (NA 1.49; Olympus). TIRF was achieved by repositioning the laser beam from the center to the rim on the back aperture of the objective using a motorized mirror. Excitation and emission light were separated via a multiband dichroic mirror (F73-866, BS R405/488/561/633; AHF Analysentechnik, Tübingen, Germany) in combination with a multiple bandpass filter (F72-866, 466/523/600/677; AHF Analysentechnik). Images were recorded with an EMCCD camera (AndoriXon DU897E; Andor, Belfast, UK) cooled to −75 °C using a pixel resolution of 512 × 512 pixels. The image from the microscope was additionally magnified via an achromatic lens (focal point 50 mm; AC254-050-A-ML; Thorlabs, Dachau/Munich, Germany). By adjusting the lens position and camera position (motorized) the pixel-size can be adjusted between 64 and 130 nm/pixel.

### dSTORM

For dSTORM measurements the 642 nm diode laser was used for excitation. Series of 4000 TIRF images (power of 642 nm laser: 141 mW, exposure time: 20 ms, pixel size: 64 nm) were recorded and subsequently analyzed for calculation of the super-resolution image. The imaging buffer for super-resolution microscopy contained sterile filtered PBS (pH 7.4) mixed 1:1 with glucose solution (0.1 g glucose, 0.9 ml PBS, 0.1 ml glycerol) and 90 mM β-mercaptoethylamine (MEA; Sigma-Aldrich, Taufkirchen, Germany; 1 M MEA-HCl in H_2_O). Imaging buffer was filled in the measurement chamber. Subsequently, 20 μl of an oxygen scavenging solution (1 mg glucose oxidase (Sigma-Aldrich), 1.2 μl catalase (from bovine liver; C100; Sigma-Aldrich), 4 mM TCEP (Sigma-Aldrich), 5.2 mM KCl, 2 mM Tris-HCl, pH 7.5, 0.5 ml glycerol, 0.45 ml H_2_O) was added. Imaging buffer was added until the chamber was completely filled and sealed with a coverslip.

### PALM

For PALM measurements in living HEK293 cells the 488 nm line of an Ar-ion laser was used for excitation at low power (<1 mW) to identify suitable cells expressing mito-Dendra2 localized in mitochondria. Subsequently, low powers of 405 nm light (less than 1 mW, adjusted during recording to assure optimal number of photoconverted mito-Dendra2 proteins absorbing at 561 nm) was used together with high power (100 mW) of 561 nm light for read out of the fluorescence of the mito-Dendra2 proteins. In this manner, sequences of 4000 TIRF images (exposure time: 50 ms; pixel size: 110 nm) were recorded and subsequently analyzed for calculation of the super-resolution image. Cells were maintained in phenol-red free Dulbecoo’s Modified Eagle Medium (DMEM with high glucose (4.5 g/ml); 21063-045 Life Technologies) and additional 10 mM HEPES buffer (pH 7.2).

## Additional Information

**How to cite this article**: Tang, Y. *et al.* SNSMIL, a real-time single molecule identification and localization algorithm for super-resolution fluorescence microscopy. *Sci. Rep.* **5**, 11073; doi: 10.1038/srep11073 (2015).

## Supplementary Material

Supplementary Information

## Figures and Tables

**Table 1:** Computing time.

	SNSMIL	rapidSTORM	QuickPALM	Localizer	MaLiang
High SNR	79	15	142	82	27
Low SNR	82	20	156	133	25
Low SNR with GB	83	27	382	135	24

Computing time of different software for three simulated datasets (unit: seconds).

All software packages were executed on the computer system as descripted in Method part. SNSMIL was running on Debian Linux 6.0 64 bit operation system whereas all other software packages were running on Windows 7 64 bit.
