# Supplementary material for: SNSMIL, a real-time single molecule identification and localization algorithm for super-resolution fluorescence microscopy
Source: Sci Rep. 2015 Jun 22;5:11073. doi: 10.1038/srep11073 (PMC4476421; doi:10.1038/srep11073)
Supplement: Supplementary Information [file srep11073-s1.pdf]

Supplementary information

SNSMIL, a real-time single molecule identification and localization algorithm for super-resolution fluorescence microscopy

Yunqing Tang[1,2,3], Luru Dai[1], Xiaoming Zhang[1], Junbai Li[1,4,*], Johnny Hendriks[5], Xiaoming Fan[5], Nadine Gruteser[5], Annika Meisenberg[5], Arnd Baumann[5], Alexandros Katranidis[6] and Thomas Gensch[5,*]

[1]*National Center for Nanoscience and Technology of China No.11, BeiyitiaoZhongguancun 100190 Beijing, China*
[2]*Department of Physics, Chongqing University, Chongqing 400044, P.R.China*
[3]*State Key Laboratory of Theoretical Physics, Institute of Theoretical Physics, Chinese Academy of Sciences, Beijing 100190, China*
[4]*Institute of Chemistry, Chinese Academy of Sciences, 100190 Beijing, P.R.China*
[5]*Institute of Complex Systems (ICS-4, Cellular Biophysics), Forschungszentrum Juelich, Leo-Brandt-Str. 52428 Juelich, Germany*
[6]*Institute of Complex Systems (ICS-5, Molecular Biophysics), Forschungszentrum Juelich, Leo-Brandt-Str. 52428 Juelich, Germany*
[*]*To whom correspondence should be addressed*

Supplementary Information

Part 1

SNSMIL Software Manual

SNSMIL Version 1.0 Manual

Yunqing Tang[1,2,3] and Luru Dai[1]

[1]*National Center for Nanoscience and Technology of China No.11, BeiyitiaoZhongguancun 100190 Beijing, China*
[2]*Department of Physics, Chongqing University, Chongqing 400044, P.R.China*
[3]*State Key Laboratory of Theoretical Physics, Institute of Theoretical Physics, Chinese Academy of Sciences, Beijing 100190, China*

Contents

1 SNSMIL (Shot Noise based Single Molecule Identification and Localization) 3
1.1 What is SNSMIL? 3
1.2 Licence 3
1.3 Hardware Requirements 3
1.4 How to Install? 3
1.5 How to Use? 4

2 Theory of Single-Molecule Localization Microscopy 8
2.1 Point Spread Function 8
2.2 The Gaussian Approximation to A Besselian PSF 8
2.3 EMCCD Theory 9
2.3.1 Correct Signal Intensity 9
2.3.2 Estimate Number of Photoelectrons 9
2.3.3 Estimate Statistical Noise 10
2.4 Rose Criterion and Quality of Spots 11
2.5 Theoretical Localization Precision 11

3 SNSMIL Algorithm 13
3.1 step1: Get Photoelectrons from Original Intensity Image 13
3.2 Step2: Find Local Brightest Spots 13
3.3 Step3: Discard Non-isolated Local Brightest Spots 13
3.4 Step4: Gaussian Smoothing 14
3.5 Step5: Estimate Local Background, CNR and Quality 14
3.6 Step6: Select by Rose Criterion and Quality 14
3.7 Step7: Estimate Initial Parameters for Gaussian PSF 15
3.8 Step8: Nonlinear Least Squares Fitting and Selecting 15
3.8.1 Levenberg-Marquardt Nonlinear Parameter Estimation 15
3.8.2 Fitting Window Size 19
3.8.3 Fitting Result Selection 19
3.9 Summary 20

A Model 22

B Localization Precision 23

Bibliography 29

Chapter 1

SNSMIL (Shot Noise based Single Molecule Identification and Localization)

1.1 What is SNSMIL?

SNSMIL is a cross-platform software aims at image analysis in photoswitching microscope including photoactivated localization microscopy (PALM) [1], stochastic optical reconstruction microscopy (STORM) [2], direct stochastic optical reconstruction microscopy (dSTORM) [3, 4], *etc.*. SNSMIL is an accurate and fast 2-dimensions and 3-dimensions (under development) real-time image analysis software based on Central Processing Unit (CPU) or Graphic Processing Unit (GPU).

1.2 Licence

SNSMIL is released under a BSD license, it is free for both academic and commercial use.

1.3 Hardware Requirements

SNSMIL GPU version needs a computer equipped Nvidia CUDA-enabled graphics card with compute capability 2.0 or higher. To determine compute capability of a Nvidia graphics card, please refer http://developer.nvidia.com/cuda/cuda-gpus.

1.4 How to Install?

We provide both a graphical user interface (GUI) version and a command line interface (CLI) version of SNSMIL. The CLI version can be embed into other programming languages (for example: LabView); We recommend that users use the SNSMIL GPU GUI version, because it is more user friendly and faster than CPU version. The SNSMIL GPU GUI version depends on CUDA runtime dynamic library, LGPL v2 Qt 4.8

dynamic library, libtiff.

a) For Windows system:
Only binary GUI version for Windows (xp & 7, 32 bit or 64 bit) system is supplied.
(1) install latest Nvidia graphic card drivers
Drivers can be found on web page http://www.nvidia.com/Download/index.aspx?lang=en-us, select the appropriate driver, download and install.
(2) install Microsoft Visual C++ 2010 Redistributable Package
For 32 bits system, that can be found on web page http://www.microsoft.com/en-us/download/details.aspx?id=5555.
For 64 bits system, that can be found on web page http://www.microsoft.com/en-us/download/details.aspx?id=14632.
(3) CUDA runtime dynamic library and LGPL v2 Qt 4.8 dynamic library
We prepare that as a separate package, users can copy all of the *.dll files to the same directory with SNSMIL.

b) For Debian Linux system:
The SNSMIL GPU GUI version depends on CUDA (4.2 or higher), Qt, libtiff.
Before installing SNSMIL GPU GUI version, users should install CUDA in accordance with the CUDA installation guide, then follow how to install SNSMIL GPU GUI version.
Steps for installing SNSMIL GPU GUI version:
(1) install software:
$aptitude install libtiff-dev build-essential libqt4-dev libqt4-dev-tools
(2) uncompress and compiled SNSMIL:
$tar -xzvf snsmil*.tar.gz
$cd snsmil*
$qmake
$make

1.5 How to Use?

Steps for using the SNSMIL GPU GUI version:
(1) File->Open, then chose a tif file.
Follow Figure 1.1 1.2.
(2) Setting->Parameters Setting, click the "Apply" button.
Optical parameters and camera parameters depend on the experiment, the parameter "Excess noise factor square" for the EMCDD camera always is 2, if the camera is a CCD camera or a CMOS camera, it should be set to 1.
Other parameters can be adjusted by users. in most case, users only need to adjust the parameter "Quality threshold".
Follow Figure 1.3 1.4.
(3) Click "show current frame result" icon, and adjust the parameter "Quality threshold" by eyes.
Follow Figure 1.5.
(4) Click "Analyse all frames" icon.
Follow Figure 1.6 1.7.

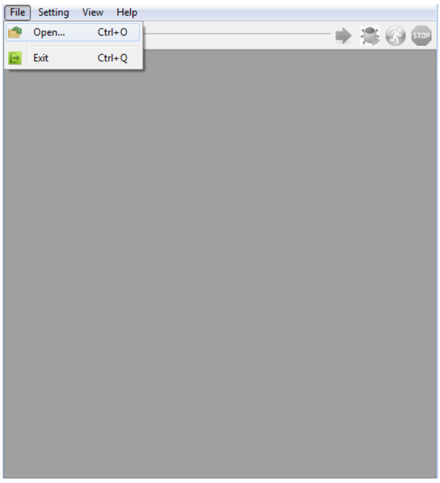

Figure 1.1: SNSMIL Step 1

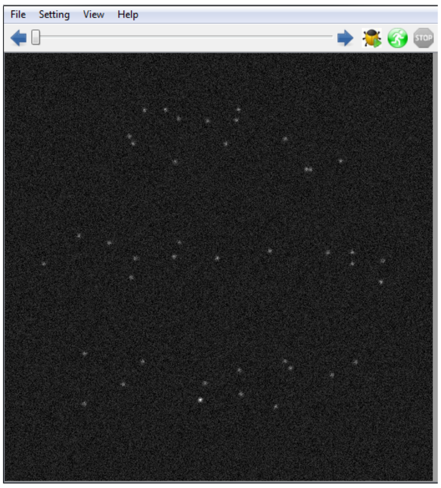

Figure 1.2: SNSMIL Step 2

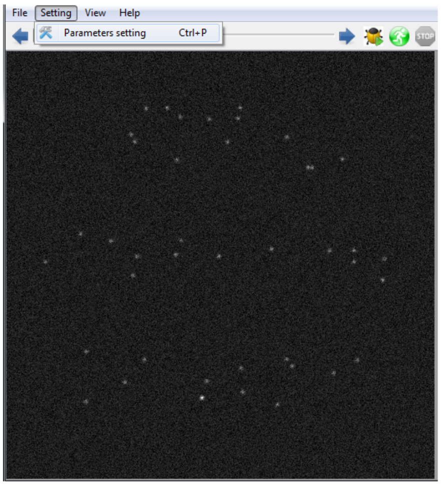

Figure 1.3: SNSMIL Step 3

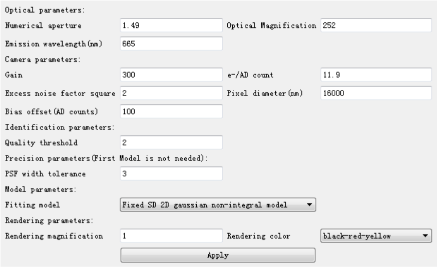

Figure 1.4: SNSMIL Step 4

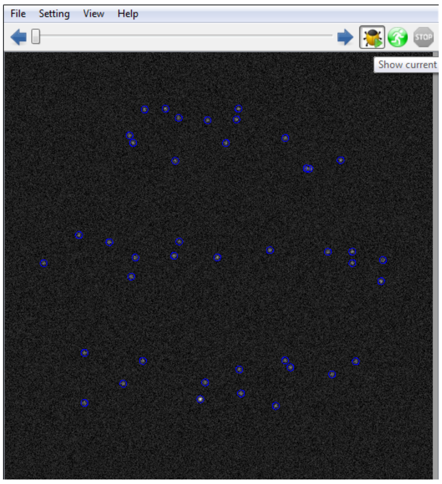

Figure 1.5: SNSMIL Step 5

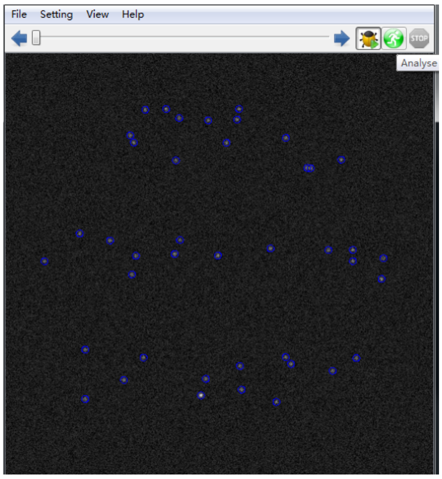

Figure 1.6: SNSMIL Step 6

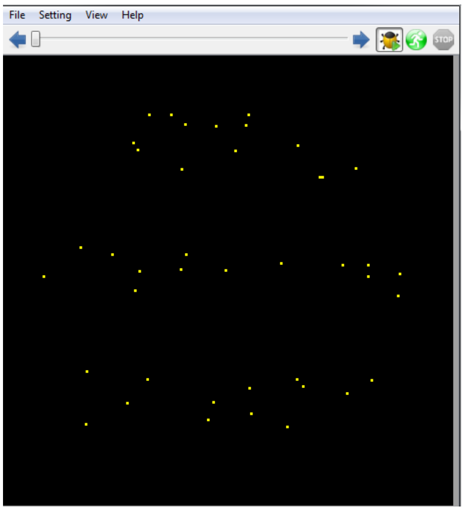

Figure 1.7: SNSMIL Step 7

Chapter 2

Theory of Single-Molecule Localization Microscopy

We assume the reader is familiar with the classical optical microscopy and fundamental of the single-molecule localization microscopy. In this chapter, we only talk about physical concepts related to localization of the single-molecule fluorescence signal.

2.1 Point Spread Function

Point spread function (PSF) of an imaging system is the intensity distribution of a image generated by a point light source passed through the system. Because the diffraction of light, the theoretical PSF is an Airy disc and given by [5]:

$$I(d) \propto (\frac{J_1(d)}{d})^2, \tag{2.1}$$

where $I(d)$ denotes the intensity in distance d from the position of the point light source in the image plane, J_1 denotes the Bessel function of the first kind and first order. The graph of the PSF is shown as figure 2.1.
The radius of the airy disc in the image plane R_{airy} is given by [6]:

$$R_{airy} = 0.61\frac{M\lambda}{\mu NA}(pixels). \tag{2.2}$$

where M denotes the magnification, λ denotes the detection wavelength, NA denotes the numerical aperture of the imaging system, in most case it defined by the objective lens, μ is the pixel size of the camera in the image plane.

2.2 The Gaussian Approximation to A Besselian PSF

As described in section 2.1, the PSF of a far field microscopy can be represented by a Bessel function. However, Bessel function is very computational intensive both for computing and fitting [7]. This difficulty can be successfully addressed using a Gaussian function that approximates the Besselian PSF [8]. Where, we use an elliptical two-dimensional (2D) Gaussian non-integral model, there are six parameters, that are

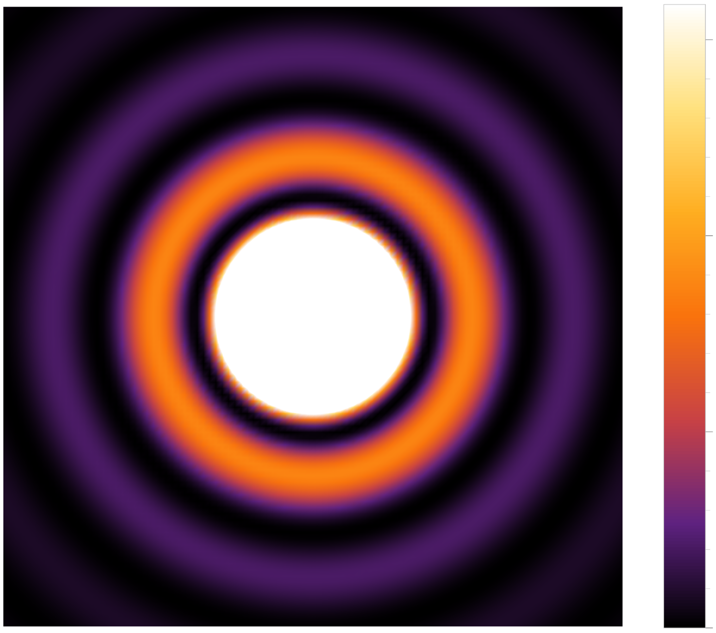

Figure 2.1: an Airy disc

$\boldsymbol{\alpha} = (n_{\gamma e^-,p}, x_0, y_0, s_x, s_y, n_{\gamma e^-,b})$, other more simplified models see the Appendix A, so the number of photoelectrons in the pixel (i, j) can be written as equation 2.3:

$$\begin{aligned} n_{\gamma e^-}(\boldsymbol{\alpha}, i, j) &= n_{\gamma e^-}(n_{\gamma e^-,p}, x_0, y_0, s_x, s_y, n_{\gamma e^-,b}, i, j) \\ &= n_{\gamma e^-,p} \exp[-\frac{(x-x_0)^2}{2s_x^2} - \frac{(y-y_0)^2}{2s_y^2}] + n_{\gamma e^-,b} \end{aligned} \tag{2.3}$$

Then the theoretical Gaussian width of the PSF is given by [9, 10]:

$$s_0 = M\lambda/(\sqrt{2}\pi\mu NA)(pixels). \tag{2.4}$$

2.3 EMCCD Theory

2.3.1 Correct Signal Intensity

To ensure that the displayed signal level is always a positive number of counts (intensity), in general, EMCCD adds I_{offset} counts for each individual image pixel [11]. for Andor iXon EMCCD, $I_{offset} = 100$ [11]. Therefore the effective signal intensity I_{eff} can be obtained by subtract the bias offset I_{offset} from intensity of a pixel I:

$$I_{eff} = \begin{cases} I - I_{offset}, & I \geq I_{offset}, \\ 0, & I < I_{offset}. \end{cases} \tag{2.5}$$

2.3.2 Estimate Number of Photoelectrons

We assume that $n_{\gamma e^-}$ is the number of photoelectrons in EMCCD camera after photons converted to photoelectrons, G_{ccd} is the EMCCD camera gain, and n_{e^-} denotes the

number of electrons after charge multiplication, we can get [11, 12]:

$$n_{e^-} = G_{ccd} n_{\gamma e^-} \tag{2.6}$$

if G_a denotes electrons/AD count, then electrons convert to digital signals I_{eff} (intensity) by [11, 12]:

$$I_{eff} = \frac{n_{e^-}}{G_a} \tag{2.7}$$

then we can estimate the number of the signal photoelectrons $n_{\gamma e^-}$:

$$n_{\gamma e^-} = \frac{n_{e^-}}{G_{ccd}} = I_{eff} \frac{G_a}{G_{ccd}} \tag{2.8}$$

For convenience, we define effective gain G_{eff} as:

$$G_{eff} = \frac{G_{ccd}}{G_a} \tag{2.9}$$

so the number of the signal photoelectrons $n_{\gamma e^-}$ can be re-written as:

$$n_{\gamma e^-} = \frac{I_{eff}}{G_{eff}} \tag{2.10}$$

2.3.3 Estimate Statistical Noise

The noise of an EMCCD camera is composed of several contributions: multiplicative noise (shot noise with excess noise factor), read-out noise, dark current. The overall noise σ can be expressed by [11, 13, 14]:

$$\sigma = \sigma_{total} = \sqrt{F^2(n_{\gamma e^-} + \sigma_{dark}^2) + (\frac{\sigma_{read}}{G_{ccd}})^2} \tag{2.11}$$

When the temperature is low, the dark current noise can be neglected [13], when the gain of the EMCCD camera is high, the read noise can be neglected, therefore, the overall noise σ can be written as:

$$\sigma = \sigma_{total} = F\sqrt{n_{\gamma e^-}} \tag{2.12}$$

where, $n_{\gamma e^-}$ denotes the number of photoelectrons, F denotes the excess noise factor of the camera, for a EMCCD camera, it has been shown that the excess noise factor tends to $\sqrt{2}$ as gain increases [14–19].

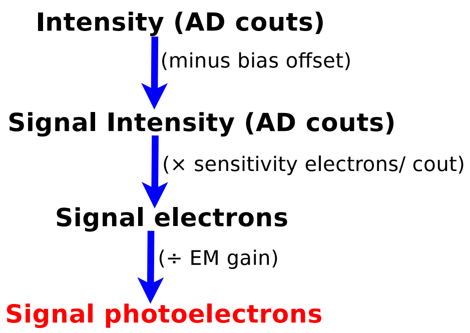

Figure 2.2: estimate the number of photoelectrons (from Andor borchure [11])

2.4 Rose Criterion and Quality of Spots

Before we talk about the Rose criterion, we define signal-to-noise ratio, the region of interest (ROI), contrast-to-noise ratio. For single pixel (i, j), the signal-to-noise ratio $\mathbf{SNR}(i, j)$ can be defined as equation 2.13:

$$\mathbf{SNR}(i,j) = \frac{n_{\gamma e^-}(i,j) - n_{\gamma e^-,b}(i,j)}{\sigma_{\gamma e^-,b}(i,j)} = \frac{n_{\gamma e^-}(i,j) - n_{\gamma e^-,b}(i,j)}{F\sqrt{n_{\gamma e^-,b}(i,j)}} \tag{2.13}$$

where, $n_{\gamma e^-}(i, j)$ denotes the number of photoelectrons of pixel (i, j), $n_{\gamma e^-,b}(i, j)$ denotes the number of background photoelectrons of pixel (i, j), $\sigma_{\gamma e^-,b}(i, j)$ denotes the noise of background of pixel (i, j), and F is the excess noise factor of the EMCCD camera.

Here, We define a ROI as a region containing the local brightest spot (i_{peak}, j_{peak}), all pixels locate within $(2ceil(s_0) + 1) \times (2ceil(s_0) + 1)$, and the $\mathbf{SNR}$ of every pixel is larger than zero, it can be shown as equation 2.14.

$$ROI = \{(i,j)|abs(i - i_{peak}) \leq ceil(s_0), abs(j - j_{peak}) \leq ceil(s_0), \mathbf{SNR}(i,j) > 0\} \tag{2.14}$$

where, s_0 is the theoretical Gaussian width of the PSF in equation 2.4, ceil() rounds the elements to the nearest greater or equal integer, abs() denotes the absolute value.

The effective signal-to-noise ratio ($\mathbf{SNR}_{eff}$) of one ROI can be defined as equation 2.15:

$$\mathbf{SNR}_{eff} = \frac{1}{N_{ROI}} \sum_{i,j}^{(i,j)\in ROI} \mathbf{SNR}(i,j) \tag{2.15}$$

where, N_{ROI} means the number of pixels of ROI, for different spots, the N_{ROI} may be not same.

Then, The effective contrast-to-noise ratio ($\mathbf{SNR}_{eff}$) of one ROI can be defined as [20–25]:

$$\mathbf{CNR}_{eff} = \sqrt{N_{ROI}}\mathbf{SNR}_{eff} \tag{2.16}$$

The Rose criterion states that a signal is readily detectable above its background noise if it reaches a level of 3-5 times the noise (in this case, we set to 3) [21–25]. This is the basis of determining the minimum contrast which can be observed.

$$\mathbf{CNR}_{eff} > 3 \tag{2.17}$$

We define the quality ($\mathbf{Q}$) of one spot as:

$$Q = \sqrt{\mathbf{SNR}_{eff} * \mathbf{CNR}_{eff}} \tag{2.18}$$

2.5 Theoretical Localization Precision

For a far field microscopy and an incoherently lighted object, the resolving power Y is given by [26]:

$$Y = 0.61\frac{\lambda}{NA}, \tag{2.19}$$

where, λ denotes the detection wavelength, NA denotes the numerical aperture of the microscope's objective.
The localization precision of the single-molecule localization microscopy in lateral

direction is given by Thompson *et al.* [27, 28]. We consider the effect of quantum efficiency (QE) and the the excess noise factor of the camera, and re-derive the two-dimensional localization precision formula follow Thompson's paper [28] (the detail of derivation see the Appendix B):

$$\langle(\Delta x_0)^2\rangle = F^2[\frac{s_x^2 + a^2/12}{n_{\gamma e^-,psf}} + \frac{8\pi(s_x^2 + a^2/12)^{\frac{3}{2}}(s_y^2 + a^2/12)^{\frac{1}{2}} n_{\gamma e^-,b}}{a^2 n_{\gamma e^-,psf}^2}] \quad (2.20)$$

$$\langle(\Delta y_0)^2\rangle = F^2[\frac{s_y^2 + a^2/12}{n_{\gamma e^-,psf}} + \frac{8\pi(s_y^2 + a^2/12)^{\frac{3}{2}}(s_x^2 + a^2/12)^{\frac{1}{2}} n_{\gamma e^-,b}}{a^2 n_{\gamma e^-,psf}^2}] \quad (2.21)$$

where, the number of all photoelectrons of the point spread function $n_{\gamma e^-,psf}$ can be calculated by equation 2.22:

$$\begin{aligned} n_{\gamma e^-,psf} &= \int_{-\infty}^{\infty} dx \int_{-\infty}^{\infty} dy\{n_{\gamma e^-,p} \exp[-\frac{(x-x_0)^2}{2s_x^2} - \frac{(y-y_0)^2}{2s_y^2}]\} \\ &= \frac{\pi n_{\gamma e^-,p} s_x s_y}{2} * [\text{erf}(\infty) - \text{erf}(-\infty)][\text{erf}(\infty) - \text{erf}(-\infty)] \\ &= 2\pi n_{\gamma e^-,p} s_x s_y \end{aligned} \quad (2.22)$$

and the $erf()$ function is the error function [29], so, the lateral distance from estimated position to real position can be written as:

$$\sqrt{\langle(\Delta r)^2\rangle} = \sqrt{\langle(\Delta x_0)^2 + (\Delta y_0)^2\rangle} = \sqrt{\langle(\Delta x_0)^2\rangle + \langle(\Delta y_0)^2\rangle} \quad (2.23)$$

is the pixel diameter of the camera, $n_{\gamma e^-,psf}$ is the number of all photoelectrons of the point spread function, s_x and s_y are the standard deviation in x direction and y direction of the point spread function, $n_{\gamma e^-,b}$ is the number of photoelectrons of the local background.

Chapter 3

SNSMIL Algorithm

SNSMIL algorithm follows two stages:
1. distinguish single-molecule signals from background (section 3.1-3.5);
2. fit every single-molecule fluorescence signal with Gaussian function (section 3.6-3.7), then get the position of every single-molecule fluorescence signal: (x_0, y_0, z_0).

3.1 step1: Get Photoelectrons from Original Intensity Image

As discussed in section 2.3.1 and 2.3.2, the number of photoelectrons $n_{\gamma e^-}(i,j)$ of every pixel can be calculated by equation 3.1:

$$n_{\gamma e^-}(i,j) = \begin{cases} (I(i,j) - I_{offset})/G_{eff}, & I \geq I_{offset}, \\ 0, \quad I < I_{offset}. \end{cases} \tag{3.1}$$

3.2 Step2: Find Local Brightest Spots

If the intensity or photoelectrons of a pixel is maximal in its neighboring region with a radius of airy disk (R_{airy}), this airy disc area is chosen as a candidate of an emitter.

3.3 Step3: Discard Non-isolated Local Brightest Spots

For convenience, we define h_f as equation 3.2:

$$h_f = ceil(R_{airy}) \tag{3.2}$$

where R_{airy} denotes the radius of airy disc with unit of number of pixel (see equation 2.2), $ceil()$ rounds the elements to the nearest greater or equal integer. If more than one brightest spots were found with the same intensity within a region with radius of airy disk (R_{airy}), we calculate the sum of the window is taken with the center of each local brightest spot (i_{peak}, j_{peak}) with window size $(2h_f + 1) \times (2h_f + 1)$, select the spot with brightest sum of the local window. In most of case, more than one brightest spots with the same intensity occur in one airy disc area indicates CCD saturation. As a matter of fact, it happens often at the beginning in a image stack and induce fake single molecule recognition. This step aims to abandon those points.

3.4 Step4: Gaussian Smoothing

Haphazardly, a shot noise signal is taken over the background. Needle-like shot noise can be distinguished from a signal emitter by implementing Gaussian smoothing on original image. We choose 3x3 mask. The Gaussian width σ is half of a pixel ($\sigma = 0.5 pixels$). This step processes in space domain. The mask is:

$$Mask(x,y) = N * \exp(-\frac{(r_x^2 + r_y^2)}{2\sigma^2}). \tag{3.3}$$

where, N is normalization coefficient, σ denotes gaussian width of the mask, r_x and r_y denotes the distance (unit:pixel) from (x, y) to the center of the 3x3 mask in x and y direction respectively. So $Mask$ can be written in matrix,

$$\begin{aligned} Mask = & \frac{1}{1 + 4\exp(-\frac{1}{\sigma^2}) + 4\exp(-\frac{1}{2\sigma^2})} * \\ & \begin{pmatrix} \exp(-\frac{1}{\sigma^2}) & \exp(-\frac{1}{2\sigma^2}) & \exp(-\frac{1}{\sigma^2}) \\ \exp(-\frac{1}{2\sigma^2}) & 1 & \exp(-\frac{1}{2\sigma^2}) \\ \exp(-\frac{1}{\sigma^2}) & \exp(-\frac{1}{2\sigma^2}) & \exp(-\frac{1}{\sigma^2}) \end{pmatrix}. \end{aligned} \tag{3.4}$$

We maintains edge pixels of $n^{(s)}_{\gamma e^-}$ the same as edge pixels of $n_{\gamma e^-}$:

$$\begin{aligned} & n^{(s)}_{\gamma e^-}(i,j) = n_{\gamma e^-}(i,j), \\ & i = 0 \text{ or } i = W-1 \text{ or } j = 0 \text{ or } j = H-1, \end{aligned} \tag{3.5}$$

and other pixels implement the gaussian smoothing:

$$\begin{aligned} & n^{(s)}_{\gamma e^-}(i,j) = \sum_{m=0,n=0}^{m=2,n=2} Mask(m,n) n_{\gamma e^-}(i+m-1, j+n-1), \\ & 0 < i < W-1 \text{ and } 0 < j < H-1. \end{aligned} \tag{3.6}$$

where, width and height of the original fluorescence image is W and H respectively. $n_{\gamma e^-}$ is the original fluorescence image (in photoelectrons) and $n^{(s)}_{\gamma e^-}$ is the image after implementing gaussian smoothing on the original photoelectrons image.

3.5 Step5: Estimate Local Background, CNR and Quality

After step 4, take a window W_1 for each local brightest spot with the size of 4 times airy disc, $(8h_f + 1) \times (8h_f + 1)$ as a mask, the values of pixels for the mask all are $\frac{1}{(8h_f+1)\times(8h_f+1)}$. Calculate the convolution between $n^{(s)}_{\gamma e^-}$ and W_1, the new image denotes of the local background for every pixel. In order to avoid the estimated local background is too small, we set the low limit of the local background to one photoelectron. Refer to 2.16 2.18 for the calculation of the CNR and the Quality.

3.6 Step6: Select by Rose Criterion and Quality

Refer to section 2.4.

3.7 Step7: Estimate Initial Parameters for Gaussian PSF

Nonlinear fitting is an iterative procedure, the algorithm must start with estimated values for each undetermined parameters. the initial values is chosen according to theoretical prediction. Gaussian width $s_x^{(0)}$ and $s_y^{(0)}$ takes theoretical value as equation 2.4:

$$s_x^{(0)} = s_0, \qquad s_y^{(0)} = s_0 \tag{3.7}$$

Initial center position is taken as (i_{peak}, j_{peak}), the local background is taken as discussed in section 3.5, and the amplitude is taken as equation 3.8.

$$n_{\gamma e^-,p} = n_{\gamma e^-}(i_{peak}, j_{peak}) - n_{\gamma e^-,b}(i_{peak}, j_{peak}) \tag{3.8}$$

3.8 Step8: Nonlinear Least Squares Fitting and Selecting

An important application of the least squares problem is parameter estimation. The least squares problem can be defined as:

Definition 1 (Least squares problem).
Let $\boldsymbol{r} : \mathbb{R}^P \to \mathbb{R}^D$ be a vector function and $P \leq D$. Find $\boldsymbol{\alpha}$, a local minimizer for

$$f(\boldsymbol{\alpha}) = \frac{1}{2}\|\boldsymbol{r}(\boldsymbol{\alpha})\|_2 = \frac{1}{2}\boldsymbol{r}(\boldsymbol{\alpha})^T\boldsymbol{r}(\boldsymbol{\alpha}) = \frac{1}{2}\sum_{d=1}^{D}(r_d(\boldsymbol{\alpha}))^2. \tag{3.9}$$

where, P denotes the number of parameters, D denotes the number of data points, $\boldsymbol{\alpha} = (\alpha_1, \ldots, \alpha_P)^T$ denote undetermined parameters, $n_{\gamma e^-}(\boldsymbol{\alpha}, i_d, j_d)$ denotes fitting model (Section 2.2 and Appendix A) that depends on parameters $\boldsymbol{\alpha}$ for given data points $(i_1, j_1, n_{\gamma e^-}(i_1, j_1)), \ldots, (i_D, j_D, n_{\gamma e^-}(i_D, j_D))$, $\|\cdot\|_2$ denotes 2-norm, and $r_d(\boldsymbol{\alpha})$ are the residuals:

$$r_d(\boldsymbol{\alpha}) = n_{\gamma e^-}(i_d, j_d) - n_{\gamma e^-}(\boldsymbol{\alpha}, i_d, j_d), \quad d = 1, \ldots, D. \tag{3.10}$$

3.8.1 Levenberg-Marquardt Nonlinear Parameter Estimation

There are several algorithms can be applied. In SNSMIL, we used Levenberg-Marquardt algorithm (LMA) [30–32]. The LMA interpolates between the Gauss-Newton algorithm (GNA) and the method of gradient descent. The LMA is more robust than the GNA, which means that in many cases it reachs a expected result even when starts from very far of the final minimum. For well-behaved functions and reasonable starting parameters, the LMA tends to be a bit slower than the GNA. LMA can also be viewed as Gauss-Newton using a trust region approach. The LMA is a popular curve-fitting algorithm used in many applications for solving generic curve-fitting problems, the step $\boldsymbol{h}$ is defined by formula 3.11:

$$\begin{aligned} (\boldsymbol{J}^T\boldsymbol{J} + \mu\boldsymbol{I})\boldsymbol{h} &= -\boldsymbol{J}^T\boldsymbol{r} \\ with \quad \boldsymbol{J} = \boldsymbol{J}(\boldsymbol{\alpha}), \boldsymbol{r} &= \boldsymbol{r}(\boldsymbol{\alpha}), \mu \geq 0. \end{aligned} \tag{3.11}$$

where, $\boldsymbol{J}$ is Jacobian, it can be calculated by formula 3.12:

$$(\boldsymbol{J}(\boldsymbol{\alpha}))_{dp} = \frac{\partial r_d(\boldsymbol{\alpha})}{\partial \alpha_p}, \quad d = 1, \ldots, D, \quad p = 1, \ldots, P. \tag{3.12}$$

During iteration, the value of μ can be updated, the updating is controlled by gain ratio:

$$\rho = \frac{\delta_f}{\delta_L}. \tag{3.13}$$

where,

$$\delta f = \frac{1}{2}(\boldsymbol{r}(\boldsymbol{\alpha}) - \boldsymbol{r}(\boldsymbol{\alpha}_{new}))^T(\boldsymbol{r}(\boldsymbol{\alpha}) + \boldsymbol{r}(\boldsymbol{\alpha}_{new})). \tag{3.14}$$

$$\delta L = L(0) - L(\boldsymbol{h}) = \frac{1}{2}\boldsymbol{h}^T(\mu\boldsymbol{h} - \boldsymbol{g}). \tag{3.15}$$

$$\boldsymbol{g} = \boldsymbol{J}(\boldsymbol{\alpha})^T\boldsymbol{r}(\boldsymbol{\alpha}). \tag{3.16}$$

where, $\boldsymbol{r}(\boldsymbol{\alpha}_{new}) = \boldsymbol{r}(\boldsymbol{\alpha}) + \boldsymbol{h}$. Follow Hans Bruun Nielsen [33], the initial value μ_0 of μ be suggested by the formula 3.17 and the formula 3.18:

$$\boldsymbol{A} = \boldsymbol{J}^T\boldsymbol{J}. \tag{3.17}$$

$$\mu_0 = \tau \cdot max_t\{A_{tt}^{[0]}\}. \tag{3.18}$$

and the stopping criteria for the algorithm be given by the formula 3.19:

$$\begin{aligned} \|\nabla f(\boldsymbol{\alpha})\|_\infty &\leq \epsilon_1, \\ \|\boldsymbol{\alpha}_{new} - \boldsymbol{\alpha}\|_2 &\leq \epsilon_2(\|\boldsymbol{\alpha}\|_2 + \epsilon_2), \\ k &\geq k_{max}. \end{aligned} \tag{3.19}$$

Fortunately, Hans Bruun Nielsen developed a open source program based matlab [33], we can recode easily it to C language as algorithm 1 or CUDA C language as algorithm 2.
We set some default values of input parameters as:
$\tau = 10^{-3}$;
$\epsilon_1 = 10^{-4}, \epsilon_2 = 10^{-8}, k_{max} = 100$; stop criteria for the algorithm.
$C_{min} = 10$. The low limit number of continuous convergence, if the number of iterations has reached or exceeded k_{max}, but the number of continuous convergence has exceeded C_{min}, we take it as a good fitting.
Output value:
$\boldsymbol{\alpha}$; parameters.
$found$, when $found == 0$, means number of iteration has reached or exceeded k_{max}, and results do not converge;
when $found == 1$ or 2, means relative error is smaller than setting value;
when $found == 3$, means the number of iterations has reached or exceeded k_{max}, but results converge.

Input: $\boldsymbol{\alpha}_0, \tau, \epsilon_1, \epsilon_2, k_{max}, C_{min}, \boldsymbol{J}(\boldsymbol{\alpha}), \boldsymbol{r}(\boldsymbol{\alpha})$

Output: $\boldsymbol{\alpha}, found$

$k := 0; v := 2; C := 0; \boldsymbol{\alpha} := \boldsymbol{\alpha}_0;$

calculate $\boldsymbol{r}(\boldsymbol{\alpha})$; $\boldsymbol{J}(\boldsymbol{\alpha})$; $\boldsymbol{g} := \boldsymbol{J}(\boldsymbol{\alpha})^T \boldsymbol{r}(\boldsymbol{\alpha})$; $\boldsymbol{A} := \boldsymbol{J}(\boldsymbol{\alpha})^T \boldsymbol{J}(\boldsymbol{\alpha})$; $found := (\|\boldsymbol{g}\|_\infty \leq \epsilon_1)$ and $\mu := \tau * max\{A_{ii}\}$

while $found == 0$ **and** $k < k_{max}$ **do**
- $k = k + 1;$
- **solve** $(\boldsymbol{A} + \mu \boldsymbol{I}) * \boldsymbol{h} = -\boldsymbol{g}$;
- calculate $\|\boldsymbol{h}\|$; $\|\boldsymbol{\alpha}\|$;
- **if** $\|\boldsymbol{h}\| < (\|\boldsymbol{\alpha}\| + \epsilon_2)\epsilon_2$ **then**
  - $found := 2;$
- **end**
- **if** $found == 0$ **then**
  - $\boldsymbol{\alpha}_{new} := \boldsymbol{\alpha} + \boldsymbol{h}$;
  - calculate $\boldsymbol{r}(\boldsymbol{\alpha}_{new})$;
  - calculate δf and δL by 3.14 and 3.15;
  - **if** $\delta f > 0$ **and** $\delta L > 0$ **then**
    - $\boldsymbol{\alpha} := \boldsymbol{\alpha}_{new};$
    - calculate $\boldsymbol{r}(\boldsymbol{\alpha})$;$\boldsymbol{J}(\boldsymbol{\alpha})$; $\boldsymbol{g} := \boldsymbol{J}(\boldsymbol{\alpha})^T \boldsymbol{r}(\boldsymbol{\alpha})$; $\boldsymbol{A} := \boldsymbol{J}(\boldsymbol{\alpha})^T \boldsymbol{J}(\boldsymbol{\alpha})$; $\|\boldsymbol{g}\|_\infty$;
    - **if** $\|\boldsymbol{g}\|_\infty \leq \epsilon_1$ **then**
      - $found := 1;$
    - **end**
    - $\mu := \mu * max(\frac{1}{3}, 1 - (2\delta f/\delta L - 1)^3); v = 2.0; C = C + 1;$
  - **else**
    - $\mu := \mu * v; v := 2 * v; C = 0;$
  - **end**
- **end**

end

if $C \geq C_{min}$ **and** $found == 0$ **then**
- found: = 3;

end

Algorithm 1: Levenberg-Marquardt algorithm for C language

Input: $\boldsymbol{\alpha}_0, \tau, \epsilon_1, \epsilon_2, k_{max}, C_{min}, \boldsymbol{J}(\boldsymbol{\alpha}), \boldsymbol{r}(\boldsymbol{\alpha})$
Output: $\boldsymbol{\alpha}, found$

$k := 0; v := 2; C := 0; \boldsymbol{\alpha} := \boldsymbol{\alpha}_0;$
copy data to GPU shared memory with multiple threads; sync threads;
calculate $\boldsymbol{r}(\boldsymbol{\alpha})$ and $\boldsymbol{J}(\boldsymbol{\alpha})$ with multiple threads; sync threads;
calculate $\boldsymbol{g} := \boldsymbol{J}(\boldsymbol{\alpha})^T \boldsymbol{r}(\boldsymbol{\alpha})$ with multiple threads; sync threads;
calculate $\boldsymbol{A} := \boldsymbol{J}(\boldsymbol{\alpha})^T \boldsymbol{J}(\boldsymbol{\alpha})$ with multiple threads; sync threads;
calculate $found := (\|\boldsymbol{g}\|_\infty \leq \epsilon_1)$ and $\mu := \tau * max\{A_{ii}\}$ with one thread;
sync threads;
while $found == 0$ **and** $k < k_{max}$ **do**
    calculate $k = k + 1$ with one thread;
    solve $(\boldsymbol{A} + \mu \boldsymbol{I}) * \boldsymbol{h} = -\boldsymbol{g}$ with one thread;
    calculate $\|\boldsymbol{h}\|$ with one thread;
    calculate $\|\boldsymbol{\alpha}\|$ with one thread;
    if $\|\boldsymbol{h}\| < (\|\boldsymbol{\alpha}\| + \epsilon_2)\epsilon_2$ **then**
        $found := 2;$
    end
    sync threads;
    if $found == 0$ **then**
        $\boldsymbol{\alpha}_{new} := \boldsymbol{\alpha} + \boldsymbol{h}$ with multiple threads; sync threads;
        calculate $\boldsymbol{r}(\boldsymbol{\alpha}_{new})$ with multiple threads; sync threads;
        calculate δf and δL by 3.14 and 3.15 with one thread; sync threads;
        if $\delta f > 0$ **and** $\delta L > 0$ **then**
            $\boldsymbol{\alpha} := \boldsymbol{\alpha}_{new}$; sync threads;
            calculate $\boldsymbol{r}(\boldsymbol{\alpha})$ and $\boldsymbol{J}(\boldsymbol{\alpha})$ with multiple threads; sync threads;
            calculate $\boldsymbol{g} := \boldsymbol{J}(\boldsymbol{\alpha})^T \boldsymbol{r}(\boldsymbol{\alpha})$ with multiple threads; sync threads;
            calculate $\boldsymbol{A} := \boldsymbol{J}(\boldsymbol{\alpha})^T \boldsymbol{J}(\boldsymbol{\alpha})$ with multiple threads; sync threads;
            calculate $\|\boldsymbol{g}\|_\infty$ with one thread;
            if $\|\boldsymbol{g}\|_\infty \leq \epsilon_1$ **then**
                $found := 1;$
            end
            $\mu := \mu * max(\frac{1}{3}, 1 - (2\delta f/\delta L - 1)^3); v = 2.0; C = C + 1$ with one thread; sync threads;
        else
            $\mu := \mu * v; v := 2 * v; C = 0$; sync threads;
        end
    end
end
if $C \geq C_{min}$ **and** $found == 0$ **then**
    found: = 3;
end

Algorithm 2: Levenberg-Marquardt algorithm for CUDA

3.8.2 Fitting Window Size

The size of fitting window is chosen the same as airy disc, the fitting window size is taken with size of $(2h_f + 1) \times (2h_f + 1)$, where,

$$h_f = ceil(R_{airy}). \tag{3.20}$$

3.8.3 Fitting Result Selection

Correct fitting results are chosen according to following conditions.
Physically, the local background and real signal are positive number, so:

$$n_{\gamma e^-,b} > 0 \tag{3.21}$$

$$n_{\gamma e^-,p} > 0 \tag{3.22}$$

Because the fluorescent emitter may defocus, therefore the Gaussian width varies, but it can not exceed experimental limit. the Gaussian width can reflect the degree of defocus, theoretically, the Gaussian width should always greater than or equal the theoretical value s_0 when the spot in the focal plane, but because the effect of the noise and the fitting error, the Gaussian width may be smaller than the theoretical value s_0. we set a low and upper thresholding factor, T_{low} and T_{upper} applied on theoretical value s_0:

$$T_{low}s_0 < |s_x| < T_{upper}s_0, T_{low}s_0 < |s_y| < T_{upper}s_0 \tag{3.23}$$

Because the finite size of the pixels due to pixelation noise, thus increases the apparent size of the emitters, and the pixelation noise is a top-hat distribution of size of one pixel, the effective Gaussian width will increase $\frac{1}{\sqrt{12}}$ (unit: pixels) [28]. In this step, we set $T_{low} = max(0.3, \frac{1}{\sqrt{12}}/s_0)$, users can use the parameter T_{upper} to adjust average localization precision.
The center position (x_0, y_0) of a fluorescent emitter after fitting can not exceed the fitting window and gives:

$$x_{low} < x_0 < x_{upper}, y_{low} < y_0 < y_{upper} \tag{3.24}$$

the real signal photoelectrons must exceed the local background noise, and at least bigger one photoelectron than the local background, so

$$n_{\gamma e^-,p} - n_{\gamma e^-,b} > F\sqrt{n_{\gamma e^-,b}} \tag{3.25}$$

$$n_{\gamma e^-,p} - n_{\gamma e^-,b} \geq 1 \tag{3.26}$$

We can set a localization precision threshold, if the precision of localization (see equation 2.20 2.21 2.23) of one signal is worse than the threshold, we throw away the signal.

$$\sqrt{\langle(\Delta r)^2\rangle} < \sigma_{threshold} \tag{3.27}$$

because the estimation of the localization precision is only a rough estimation, that is not very accurate, we can not put it as an important criterion, in general, the localization precision is better than the resolution of the conventional microscopy, so we choose $\sigma_{threshold} = R_{airy}$.
Last, we need consider that the fitting is good or not good, coefficient of determination R^2 is a statistic that denotes the goodness of fit of a model, for nonlinear models there

is no universal R^2, one of definitions from the reference [34], and shown as equation 3.28:

$$R^2_{COR} = \widehat{COR}^2 [n_{\gamma e^-}(i,j), n_{\gamma e^-}(\boldsymbol{\alpha}, i, j)] \tag{3.28}$$

where, $\widehat{COR}[]$ is Pearson's correlation coefficient, it is a statistical measure the similarity between the experimental data and the fitting data, and it always between -1 and 1, in this case, it should always between 0 to 1, a large correlation coefficients means stronger relationships, it is defined as [35]:

$$R_{COR} = \widehat{COR}[A, B] = \frac{\sum_{k=1}^{n} (A_k - \bar{A})(B_k - \bar{B})}{\sqrt{\sum_{k=1}^{n} (A_k - \bar{A})^2} \sqrt{\sum_{k=1}^{n} (B_k - \bar{B})^2}} \tag{3.29}$$

but do not using the goodness of fit as main criterion [36]. For convenience, SNSMIL defines goodness of fit as R_{COR} since it runs monotonically to its square R^2_{COR} in the range of 0 to 1. Then we set a low threshold for Pearson's correlation coefficient:

$$R_{COR} > R_{low} \tag{3.30}$$

Typically, we recommend $R_{low} = 0.3$.

3.9 Summary

We shown SNSMIL with a flow clearly as:

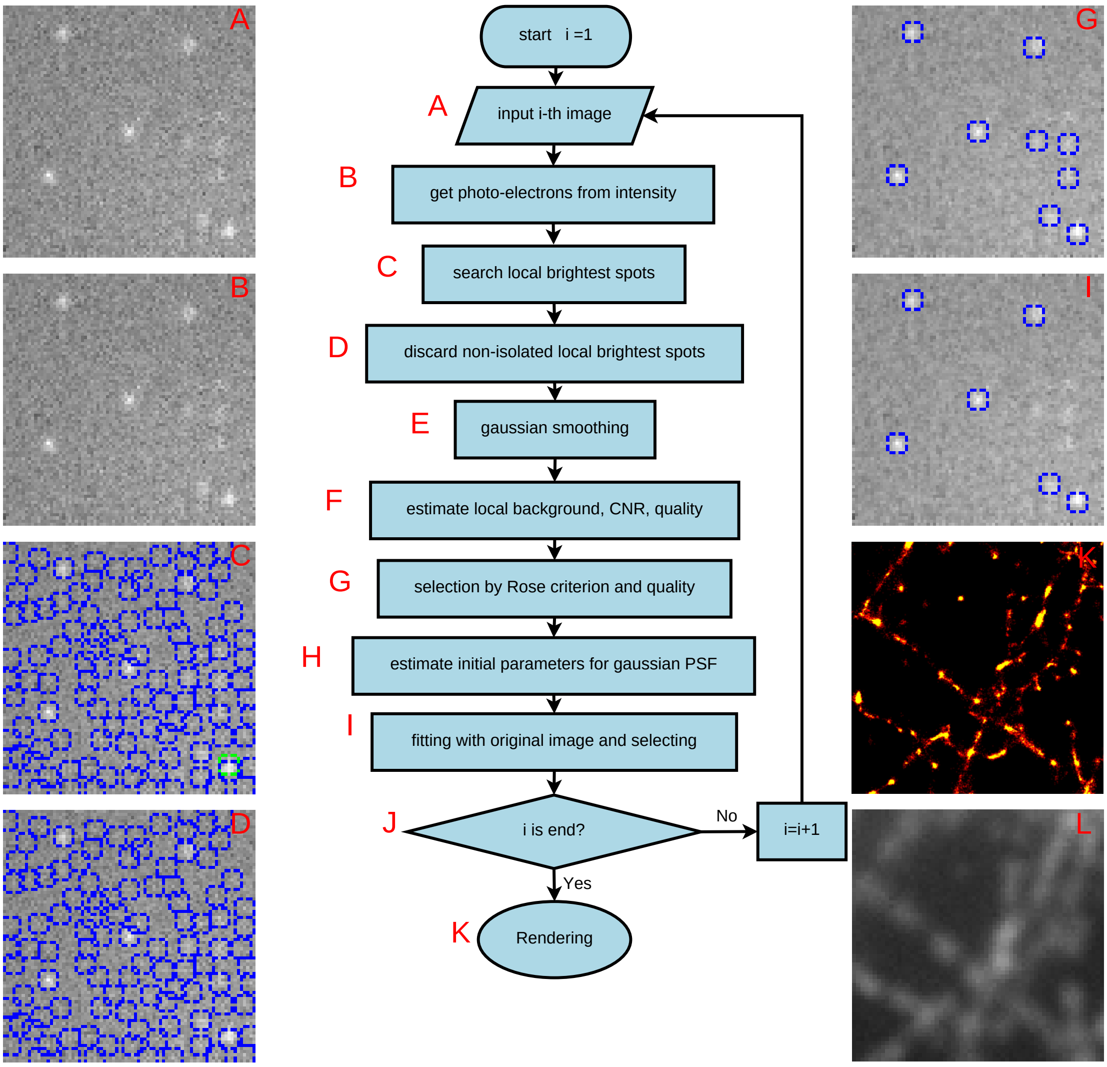

Figure 3.1: Schematic diagram of SNSMIL work flow. (K) super-resolution image, (L) TIRF image.

Appendix A

Model

fixed SD 2D Gaussian Non-integral Model:

$$\begin{aligned} n_{\gamma e^-}(\boldsymbol{\alpha}, i, j) &= n_{\gamma e^-}(n_{\gamma e^-,p}, x_0, y_0, n_{\gamma e^-,b}, i, j) \\ &= n_{\gamma e^-,p} \exp[-\frac{(i-x_0)^2 + (j-y_0)^2}{2s_0^2}] + n_{\gamma e^-,b} \end{aligned} \tag{A.1}$$

Symmetrical 2D Gaussian Non-integral Model:

$$\begin{aligned} n_{\gamma e^-}(\boldsymbol{\alpha}, i, j) &= n_{\gamma e^-}(n_{\gamma e^-,p}, x_0, y_0, s, n_{\gamma e^-,b}, i, j) \\ &= n_{\gamma e^-,p} \exp[-\frac{(i-x_0)^2 + (j-y_0)^2}{2s^2}] + n_{\gamma e^-,b} \end{aligned} \tag{A.2}$$

Elliptical 2D Gaussian Non-integral Model:

$$\begin{aligned} n_{\gamma e^-}(\boldsymbol{\alpha}, i, j) &= n_{\gamma e^-}(n_{\gamma e^-,p}, x_0, y_0, s_x, s_y, n_{\gamma e^-,b}, i, j) \\ &= n_{\gamma e^-,p} \exp[-\frac{(i-x_0)^2}{2s_x^2} - \frac{(j-y_0)^2}{2s_y^2}] + n_{\gamma e^-,b} \end{aligned} \tag{A.3}$$

Appendix B

Localization Precision

For the convenience, we start from elliptical 2D Gaussian non-integral model (see Appendix A),

$$\begin{aligned} n_{\gamma e^-}(\boldsymbol{\alpha}, i, j) &= n_{\gamma e^-}(n_{\gamma e^-,p}, x_0, y_0, s_x, s_y, n_{\gamma e^-,b}, i, j) \\ &= n_{\gamma e^-,p} \exp[-\frac{(i-x_0)^2}{2s_x^2} - \frac{(j-y_0)^2}{2s_y^2}] + n_{\gamma e^-,b} \end{aligned} \tag{B.1}$$

and, the sum of squared errors can be written as:

$$\chi^2(\boldsymbol{\alpha}) = \sum_{i,j} \frac{(n_{\gamma e^-}(i,j) - n_{\gamma e^-}(\boldsymbol{\alpha}, i, j))^2}{\sigma^2(\boldsymbol{\alpha}, i, j)}. \tag{B.2}$$

the noise $\sigma(\boldsymbol{\alpha}, i, j)$ of the pixel (i, j) can be written as (see equation 2.12):

$$\sigma^2(\boldsymbol{\alpha}, i, j) = F^2 \sigma^2_{shot}(\boldsymbol{\alpha}, i, j) = F^2 n_{\gamma e^-}(\boldsymbol{\alpha}, i, j). \tag{B.3}$$

we set:

$$\Delta n_{i,j} = n_{\gamma e^-}(n_{\gamma e^-,p}, x_0^{(real)}, y_0, s_x, s_y, n_{\gamma e^-,b}, i, j) - n_{\gamma e^-}(i, j). \tag{B.4}$$

so, the expectation and the variance of $\Delta n_{i,j}$ can be expressed as:

$$\langle \Delta n_{i,j} \rangle = 0. \tag{B.5}$$

$$\langle (\Delta n_{i,j})^2 \rangle = \sigma^2(\boldsymbol{\alpha}, i, j). \tag{B.6}$$

In order to minimize $\chi^2(\boldsymbol{\alpha})$, so:

$$\frac{\partial \chi^2(\boldsymbol{\alpha})}{\partial x_0} = 0. \tag{B.7}$$

from equation B.7

$$\begin{aligned} \frac{\partial \chi^2(\boldsymbol{\alpha})}{\partial x_0} =& \sum_{i,j} \frac{2[n_{\gamma e^-}(\boldsymbol{\alpha}, i, j) - n_{\gamma e^-}(i,j)] \frac{\partial n_{\gamma e^-}(\boldsymbol{\alpha}, i, j)}{\partial x_0} \sigma^2(\boldsymbol{\alpha}, i, j)}{\sigma^4(\boldsymbol{\alpha}, i, j)} \\ &- \sum_{i,j} \frac{2[n_{\gamma e^-}(\boldsymbol{\alpha}, i, j) - n_{\gamma e^-}(i,j)]^2 \sigma(\boldsymbol{\alpha}, i, j) \frac{\partial \sigma(\boldsymbol{\alpha}, i, j)}{\partial x_0}}{\sigma^4(\boldsymbol{\alpha}, i, j)} = 0. \end{aligned} \tag{B.8}$$

so we get:

$$\sum_{i,j} \frac{[n_{\gamma e^-}(\boldsymbol{\alpha}, i, j) - n_{\gamma e^-}(i, j)] \frac{\partial n_{\gamma e^-}(\boldsymbol{\alpha}, i, j)}{\partial x_0}}{\sigma^2(\boldsymbol{\alpha}, i, j)} = \sum_{i,j} \frac{[n_{\gamma e^-}(\boldsymbol{\alpha}, i, j) - n_{\gamma e^-}(i, j)]^2 \frac{\partial \sigma(\boldsymbol{\alpha}, i, j)}{\partial x_0}}{\sigma^3(\boldsymbol{\alpha}, i, j)}. \tag{B.9}$$

because $n_{\gamma e^-}(\boldsymbol{\alpha}, i, j)$ can be expressed as:

$$n_{\gamma e^-}(\boldsymbol{\alpha}, i, j) = n_{\gamma e^-}(n_{\gamma e^-,p}, x_0^{(real)}, y_0, s_x, s_y, n_{\gamma e^-,b}, i, j) + \frac{\partial n_{\gamma e^-}(\boldsymbol{\alpha}, i, j)}{\partial x_0} \Delta x_0. \tag{B.10}$$

so, from equation B.4 and B.10, we can get:

$$n_{\gamma e^-}(\boldsymbol{\alpha}, i, j) - n_{\gamma e^-}(i, j) = \Delta n_{i,j} + \frac{\partial n_{\gamma e^-}(\boldsymbol{\alpha}, i, j)}{\partial x_0} \Delta x_0. \tag{B.11}$$

put equation B.11 into equation B.9,

$$\sum_{i,j} \frac{[\Delta n_{i,j} + \frac{\partial n_{\gamma e^-}(\boldsymbol{\alpha}, i, j)}{\partial x_0} \Delta x_0] \frac{\partial n_{\gamma e^-}(\boldsymbol{\alpha}, i, j)}{\partial x_0}}{\sigma^2(\boldsymbol{\alpha}, i, j)} = \sum_{i,j} \frac{[\Delta n_{i,j} + \frac{\partial n_{\gamma e^-}(\boldsymbol{\alpha}, i, j)}{\partial x_0} \Delta x_0]^2 \frac{\partial \sigma(\boldsymbol{\alpha}, i, j)}{\partial x_0}}{\sigma^3(\boldsymbol{\alpha}, i, j)}. \tag{B.12}$$

and we neglect the term that contains ${\Delta x_0}^2$, then, we obtain:

$$\begin{aligned} &\sum_{i,j} \frac{[\Delta n_{i,j} + \frac{\partial n_{\gamma e^-}(\boldsymbol{\alpha}, i, j)}{\partial x_0} \Delta x_0] \frac{\partial n_{\gamma e^-}(\boldsymbol{\alpha}, i, j)}{\partial x_0}}{\sigma^2(\boldsymbol{\alpha}, i, j)} \\ &= \sum_{i,j} \frac{[\Delta n_{i,j} + \frac{\partial n_{\gamma e^-}(\boldsymbol{\alpha}, i, j)}{\partial x_0} \Delta x_0]^2 \frac{\partial \sigma(\boldsymbol{\alpha}, i, j)}{\partial x_0}}{\sigma^3(\boldsymbol{\alpha}, i, j)} \\ &\approx \sum_{i,j} \frac{[(\Delta n_{i,j})^2 + 2\Delta n_{i,j} \frac{\partial n_{\gamma e^-}(\boldsymbol{\alpha}, i, j)}{\partial x_0} \Delta x_0] \frac{\partial \sigma(\boldsymbol{\alpha}, i, j)}{\partial x_0}}{\sigma^3(\boldsymbol{\alpha}, i, j)}. \end{aligned} \tag{B.13}$$

so,

$$\begin{aligned} &\Delta x_0 \sum_{i,j} \{ \frac{[\frac{\partial n_{\gamma e^-}(\boldsymbol{\alpha}, i, j)}{\partial x_0}]^2}{\sigma^2(\boldsymbol{\alpha}, i, j)} - \frac{2\Delta n_{i,j} \frac{\partial \sigma(\boldsymbol{\alpha}, i, j)}{\partial x_0} \frac{\partial n_{\gamma e^-}(\boldsymbol{\alpha}, i, j)}{\partial x_0}}{\sigma^3(\boldsymbol{\alpha}, i, j)} \} \\ &= \sum_{i,j} \{ \frac{(\Delta n_{i,j})^2 \frac{\partial \sigma(\boldsymbol{\alpha}, i, j)}{\partial x_0}}{\sigma^3(\boldsymbol{\alpha}, i, j)} - \frac{\Delta n_{i,j} \frac{\partial n_{\gamma e^-}(\boldsymbol{\alpha}, i, j)}{\partial x_0}}{\sigma^2(\boldsymbol{\alpha}, i, j)} \}. \end{aligned} \tag{B.14}$$

and form equation B.3, we can get:

$$2\sigma(\boldsymbol{\alpha}, i, j) \frac{\partial \sigma(\boldsymbol{\alpha}, i, j)}{\partial x_0} = F^2 \frac{\partial n_{\gamma e^-}(\boldsymbol{\alpha}, i, j)}{\partial x_0}. \tag{B.15}$$

so, we obtain:

$$\begin{aligned} &\Delta x_0 \sum_{i,j} \{ \frac{[\frac{\partial n_{\gamma e^-}(\boldsymbol{\alpha}, i, j)}{\partial x_0}]^2}{\sigma^2(\boldsymbol{\alpha}, i, j)} - \frac{F^2 \Delta n_{i,j} [\frac{\partial n_{\gamma e^-}(\boldsymbol{\alpha}, i, j)}{\partial x_0}]^2}{\sigma^4(\boldsymbol{\alpha}, i, j)} \} \\ &= \sum_{i,j} \{ \frac{F^2 (\Delta n_{i,j})^2 \frac{\partial n_{\gamma e^-}(\boldsymbol{\alpha}, i, j)}{\partial x_0}}{2\sigma^4(\boldsymbol{\alpha}, i, j)} - \frac{\Delta n_{i,j} \frac{\partial n_{\gamma e^-}(\boldsymbol{\alpha}, i, j)}{\partial x_0}}{\sigma^2(\boldsymbol{\alpha}, i, j)} \}. \end{aligned} \tag{B.16}$$

because $\frac{\Delta n_{i,j}}{\sigma^2(\boldsymbol{\alpha},i,j)} \ll 1$, so we neglect the term, then, obtain:

$$\begin{aligned}\Delta x_0 &= -\frac{\sum_{i,j} \frac{\Delta n_{i,j}\frac{\partial n_{\gamma e^-}(\boldsymbol{\alpha},i,j)}{\partial x_0}}{\sigma^2(\boldsymbol{\alpha},i,j)}(1-\frac{F^2\Delta n_{i,j}}{2\sigma^2(\boldsymbol{\alpha},i,j)})}{\sum_{i,j}\frac{[\frac{\partial n_{\gamma e^-}(\boldsymbol{\alpha},i,j)}{\partial x_0}]^2}{\sigma^2(\boldsymbol{\alpha},i,j)}(1-\frac{F^2\Delta n_{i,j}}{\sigma^2(\boldsymbol{\alpha},i,j)})} \\ &\approx -\frac{\sum_{i,j}\frac{\Delta n_{i,j}\frac{\partial n_{\gamma e^-}(\boldsymbol{\alpha},i,j)}{\partial x_0}}{\sigma^2(\boldsymbol{\alpha},i,j)}}{\sum_{i,j}\frac{[\frac{\partial n_{\gamma e^-}(\boldsymbol{\alpha},i,j)}{\partial x_0}]^2}{\sigma^2(\boldsymbol{\alpha},i,j)}}.\end{aligned} \tag{B.17}$$

then, we get $\langle(\Delta x_0)^2\rangle$:

$$\begin{aligned}\langle(\Delta x_0)^2\rangle &= \langle\{\frac{\sum_{i,j}\frac{\Delta n_{i,j}\frac{\partial n_{\gamma e^-}(\boldsymbol{\alpha},i,j)}{\partial x_0}}{\sigma^2(\boldsymbol{\alpha},i,j)}}{\sum_{i,j}\frac{[\frac{\partial n_{\gamma e^-}(\boldsymbol{\alpha},i,j)}{\partial x_0}]^2}{\sigma^2(\boldsymbol{\alpha},i,j)}}\}^2\rangle \\ &= \langle\frac{\sum_{i,j}\frac{\Delta n_{i,j}\frac{\partial n_{\gamma e^-}(\boldsymbol{\alpha},i,j)}{\partial x_0}}{\sigma^2(\boldsymbol{\alpha},i,j)}\sum_{p,q}\frac{\Delta n_{p,q}\frac{\partial n_{\gamma e^-}(\boldsymbol{\alpha},p,q)}{\partial x_0}}{\sigma^2(\boldsymbol{\alpha},p,q)}}{[\sum_{i,j}\frac{[\frac{\partial n_{\gamma e^-}(\boldsymbol{\alpha},i,j)}{\partial x_0}]^2}{\sigma^2(\boldsymbol{\alpha},i,j)}]^2}\rangle \\ &= \frac{\sum_{i,j,p,q}\frac{\langle\Delta n_{i,j}\Delta n_{p,q}\rangle\frac{\partial n_{\gamma e^-}(\boldsymbol{\alpha},i,j)}{\partial x_0}\frac{\partial n_{\gamma e^-}(\boldsymbol{\alpha},p,q)}{\partial x_0}}{\sigma^2(\boldsymbol{\alpha},i,j)\sigma^2(\boldsymbol{\alpha},p,q)}}{[\sum_{i,j}\frac{[\frac{\partial n_{\gamma e^-}(\boldsymbol{\alpha},i,j)}{\partial x_0}]^2}{\sigma^2(\boldsymbol{\alpha},i,j)}]^2}.\end{aligned} \tag{B.18}$$

because $\langle\Delta n_{i,j}\Delta n_{p,q}\rangle = \delta_{i,p}\delta_{p,q}\langle(\Delta n_{i,j})^2\rangle = \sigma^2(\boldsymbol{\alpha},i,j)$, and we replace sum with an integration, so:

$$\begin{aligned}\langle(\Delta x_0)^2\rangle &= \frac{1}{\sum_{i,j}\frac{[\frac{\partial n_{\gamma e^-}(\boldsymbol{\alpha},i,j)}{\partial x_0}]^2}{\sigma^2(\boldsymbol{\alpha},i,j)}} = \frac{1}{\sum_{i,j}\frac{[\frac{\partial n_{\gamma e^-}(\boldsymbol{\alpha},i,j)}{\partial x_0}]^2}{F^2 n_{\gamma e^-}(\boldsymbol{\alpha},i,j)}} \\ &= \frac{1}{\int_{-\infty}^{\infty}di\int_{-\infty}^{\infty}dj\frac{[\frac{\partial n_{\gamma e^-}(\boldsymbol{\alpha},i,j)}{\partial x_0}]^2}{F^2 n_{\gamma e^-}(\boldsymbol{\alpha},i,j)}}.\end{aligned} \tag{B.19}$$

and, the number of photoelectrons of the PSF can calculate by:

$$\begin{aligned}n_{\gamma e^-,psf} &= \int_{-\infty}^{\infty}di\int_{-\infty}^{\infty}dj\{n_{\gamma e^-,p}\exp[-\frac{(i-x_0)^2}{2s_x^2}-\frac{(j-y_0)^2}{2s_y^2}]\} \\ &= 2\pi n_{\gamma e^-,p}s_x s_y.\end{aligned} \tag{B.20}$$

now, we consider two limits of the signal noise and the background noise, in the case of pure signal,

$$\begin{aligned}\langle(\Delta x_0)^2\rangle &= \frac{1}{\int_{-\infty}^{\infty}di\int_{-\infty}^{\infty}dj\frac{[\frac{\partial n_{\gamma e^-}(\boldsymbol{\alpha},i,j)}{\partial x_0}]^2}{F^2 n_{\gamma e^-,p}\exp[-\frac{(i-x_0)^2}{2s_x^2}-\frac{(j-y_0)^2}{2s_y^2}]}} \\ &= \frac{F^2 s_x^2}{n_{\gamma e^-,psf}}.\end{aligned} \tag{B.21}$$

in the case of pure background, to make the units correct, the pixel size a should be inserted.

$$\begin{aligned}\langle(\Delta x_0)^2\rangle &= \frac{1}{a^2\int_{-\infty}^{\infty} di \int_{-\infty}^{\infty} dj \frac{[\frac{\partial n_{\gamma e^-}(\boldsymbol{\alpha},i,j)}{\partial x_0}]^2}{F^2 n_{\gamma e^-,b}}} \\ &= \frac{8F^2\pi s_x^3 s_y n_{\gamma e^-,b}}{a^2 n_{\gamma e^-,psf}^2}.\end{aligned} \tag{B.22}$$

$$\langle(\Delta x_0)^2\rangle = \frac{F^2 s_x^2}{n_{\gamma e^-,psf}} + \frac{8F^2\pi s_x^3 s_y n_{\gamma e^-,b}}{a^2 n_{\gamma e^-,psf}^2}. \tag{B.23}$$

because the effect of the finite pixel size, it causes the pixelation noise [28], the standard deviation of the PSF should be corrected as:

$$s_x^* = s_x^2 + a^2/12, \quad s_y^* = s_y^2 + a^2/12. \tag{B.24}$$

$$\langle(\Delta x_0)^2\rangle = \frac{F^2 s_x^{*2}}{n_{\gamma e^-,psf}} + \frac{8F^2\pi s_x^{*3} s_y^* n_{\gamma e^-,b}}{a^2 n_{\gamma e^-,psf}^2}. \tag{B.25}$$

similarly, the $\langle(\Delta y_0)^2\rangle$ can be written as:

$$\langle(\Delta y_0)^2\rangle = \frac{F^2 s_y^{*2}}{n_{\gamma e^-,psf}} + \frac{8F^2\pi s_y^{*3} s_x^* n_{\gamma e^-,b}}{a^2 n_{\gamma e^-,psf}^2}. \tag{B.26}$$

Bibliography

[1] Betzig, E. *et al.* Imaging Intracellular Fluorescent Proteins at Nanometer Resolution. *Science* **313**, 1642-1645 (2006).

[2] Huang, B. *et al.* Three-Dimensional Super-Resolution Imaging by Stochastic Optical Reconstruction microscope. *Science* **319**, 810-813 (2008).

[3] Schuttpelz, M., Wolter, S., van de Linde, S., Heilemann, M. & Sauer, M. dSTORM: Real-time subdiffraction-resolution fluorescence imaging with organic fluorophores. *Proc. SPIE* **7571**, 75710V (2010).

[4] van de Linde, S. *et al.* Direct stochastic optical reconstruction microscopy with standard fluorescent probes. *Nat. Protocols* **6**, 991-1009 (2011).

[5] Heilemann, M. Design of single-molecule optical devices: unidirectional photonic wires and digital photoswitches. *Ph.D. Thesis, Bielefeld University* (2005).

[6] Amos, W. B. *Protein Localization by Fluorescence Microscopy: A Practical Approach 1st edn,* (eds Allan, V. J.) Ch. 4, 67-108 (Oxford University Press, 2000).

[7] Karatsuba, C. A. Fast evaluation of bessel functions. *Integral Transforms Spec. Funct.* **1**, 269-276 (1993).

[8] Santos, A. & Young, I. T. Model-based resolution: Applying the theory in quantitative microscopy. *Appl. Opt.* **39**, 2948-2958 (2000).

[9] Zhang, B., Zerubia, J. & Olivo-Marin, J. C. Gaussian approximations of fluorescence microscope point-spread function models. *Appl. Opt.* **46**, 1819-1829 (2007).

[10] Henriques, R. *et al.* QuickPALM: 3D real-time photoactivation nanoscopy image processing in ImageJ. *Nat. Methods* **7**, 339-340 (2010).

[11] Andor Technology Ltd. *Andor iXon3 EMCCD Brochure, Technical Note, page 35, 52-53.* (2014) Available at: https://www.andor.com/pdfs/literature/Andor_iXon3_EMCCD_Brochure.pdf. (Accessed: 10th December 2014)

[12] DeWeert, M. J., Cole, J. B., Sparks, A. W. & Acker, A. Photon transfer methods and result for electron multiplication CCDs. *Proc. SPIE* **5558**, 248-259 (2004).

[13] Soesbe, T. C., Lewis, M. A., Slavine, N. V. & Antich, P. P. Development and Evaluation of an EMCCD Based Gamma Camera for Preclinical SPECT Imaging. *IEEE Trans. Nucl. Sci.* **54**, 1516-1524 (2007).

[14] Robbins, M. S. & Hadwen, B. J. The noise performance of electron multiplying charge-coupled devices. *IEEE Trans. Electron. Dev.* **50**, 1227-1232 (2003).

[15] Daigle, O. *et al*. Extreme faint flux imaging with an EMCCD. *Publ. Astron. Soc. Pac.* **121**, 866-884 (2009).

[16] Hynecek, J. Impactron-A new solid state image intensifier. *IEEE Trans. Electron. Dev.* **48**, 2238-2241 (2001).

[17] Matsuo, K., Teich, M. C. & Saleh, B. E. A. Noise properties and time response of the staircase avalanche photodiode. *IEEE Trans. Electron. Dev.* **32**, 2615-2623 (1985).

[18] Hollenhorst, J. N. A theory of multiplication noise. *IEEE Trans. Electron. Dev.* **37**, 781-788 (1990).

[19] Denvir, D. J. & Conroy, E. Electron multiplying CCDs. *Proc. SPIE* **4877**, 55-68 (2003).

[20] Bao, Q. & Chatziioannou, A. F. Estimation of the minimum detectable activity of preclinical PET imaging systems with an analytical method. *Med. Phys.* **37**, 6070-6083 (2010).

[21] Cherry, S. R., Sorenson, J. A. & Phelps, M. E. *Physics in Nuclear Medicine 3rd edn*, Ch. 15, 264-290 (Elsevier Health Sciences, 2003).

[22] Rose, A. The sensitivity of the human eye on an absolute scale. *J. Opt. Soc. Am. A* **38**, 196 (1948).

[23] Hendrick, R. E. *Breast MRI Fundamentals and Technical Aspects 1st edn*, Ch. 7, 108 (Springer, 2008).

[24] Cunningham, A. I. & Shaw, R. Signal-to-noise optimization of medical imaging systems. *J. Opt. Soc. Am. A* **16**, 621-632 (1999).

[25] Burgess, A. The Rose model, revisited. *J. Opt. Soc. Am. A* **16**, 633 (1999).

[26] Born, M. & Wolf, E. *Principles of Optics: Electromagnetic Theory of Propagation, Interference and Diffraction of Light 6th edn*, Ch. 8, 419 (Cambridge University Press, 1980).

[27] Yildiz, A. *et al*. Myosin V Walks Hand-Over-Hand: Single Fluorophore Imaging with 1.5-nm Localization. *Science* **300**, 2061-2065 (2003).

[28] Thompson, R. E., Larson, D. R. & Webb, W. W. Precise Nanometer Localization Analysis for Individual Fluorescent Probes. *Biophys. J.* **82**, 2775-2783 (2002).

[29] Temme, N. M. *NIST Handbook of Mathematical Functions 1st edn,* (eds Olver, F. W. J. *et al.*) Ch. 7, 159-170 (Cambridge University Press, 2010).

[30] Levenberg, K. A Method for the Solution of Certain Non-Linear Problems in Least Squares. *Quart. J. Appl. Math.* **2**, 164-168 (1944).

[31] Marquardt, D. W. An Algorithm for Least-Squares Estimation of Nonlinear Parameters. *J. Soc. Indust. Appl. Math.* **11**, 431-441 (1963).

[32] Madsen, K., Nielsen, H. B. & Tingleff, O. *Methods for non-linear least squares problems 2nd edn*, Ch. 3, 24-28 (Technical University of Denmark, 2004).

[33] Nielsen, H. B. & Madsen, K. *Introduction to Optimization and Data Fitting*. (2014) Available at: http://www2.imm.dtu.dk/˜hbni/immoptibox/. (Accessed: 10th December 2014)

[34] Cameron, A. C. & Trivedi, P. K. *Microeconometrics: Methods and Applications 1st edn*, Ch. 8, 288 (Cambridge University Press, 2005).

[35] Rodgers, J. L. & Nicewander, A. W. Thirteen Ways to Look at the Correlation Coefficient. *American Statistician* **42**, 59-66 (1988).

[36] Motulsky, H. & Christopoulos, A. *Fitting Models to Biological Data using Linear and Nonlinear Regression: A practical guide to curve fitting 1st edn*, Ch. 1, 34 (Oxford University Press, 2004).

Supplementary Information
Part 2

Software Settings Used for Performance Evaluation in Results Part

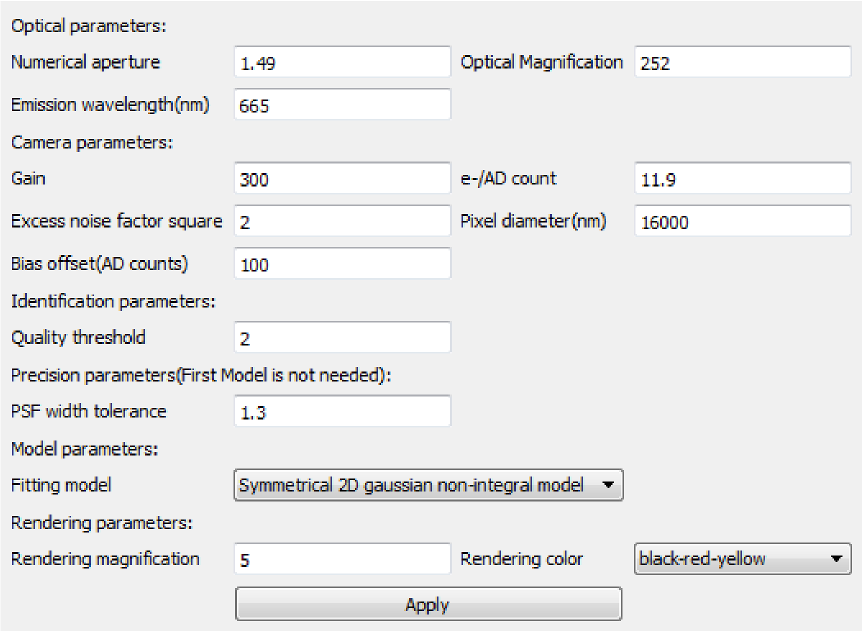

Figure S1: First parameters setting in SNSMIL (high SNR)

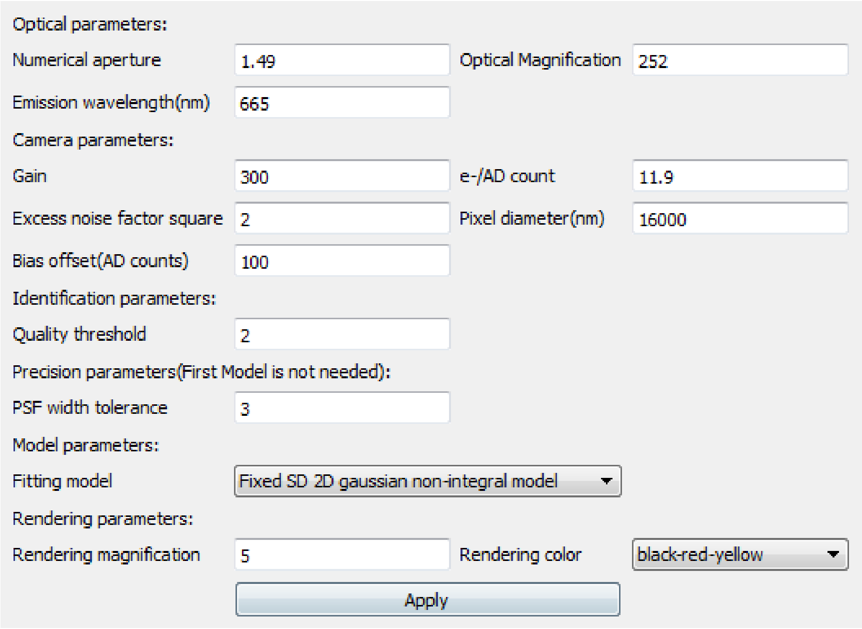

Figure S2: Second parameters setting in SNSMIL (high SNR)

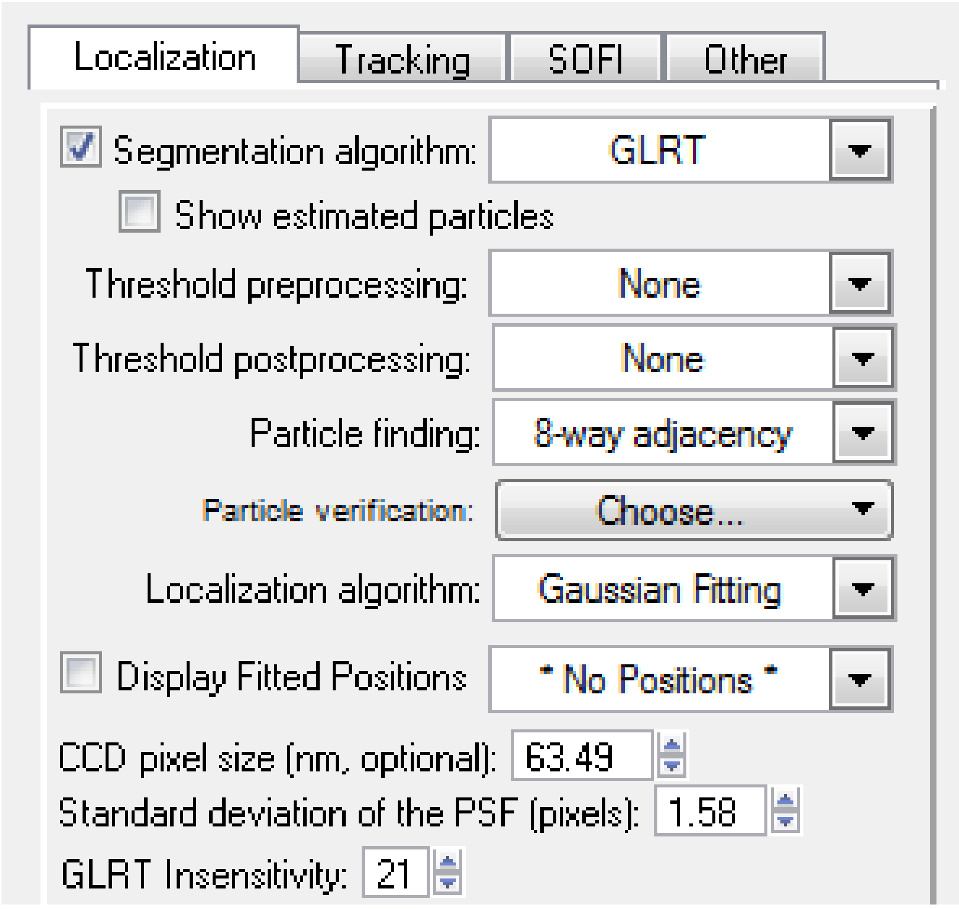

Figure S3: Parameters setting in Localizer (high SNR)

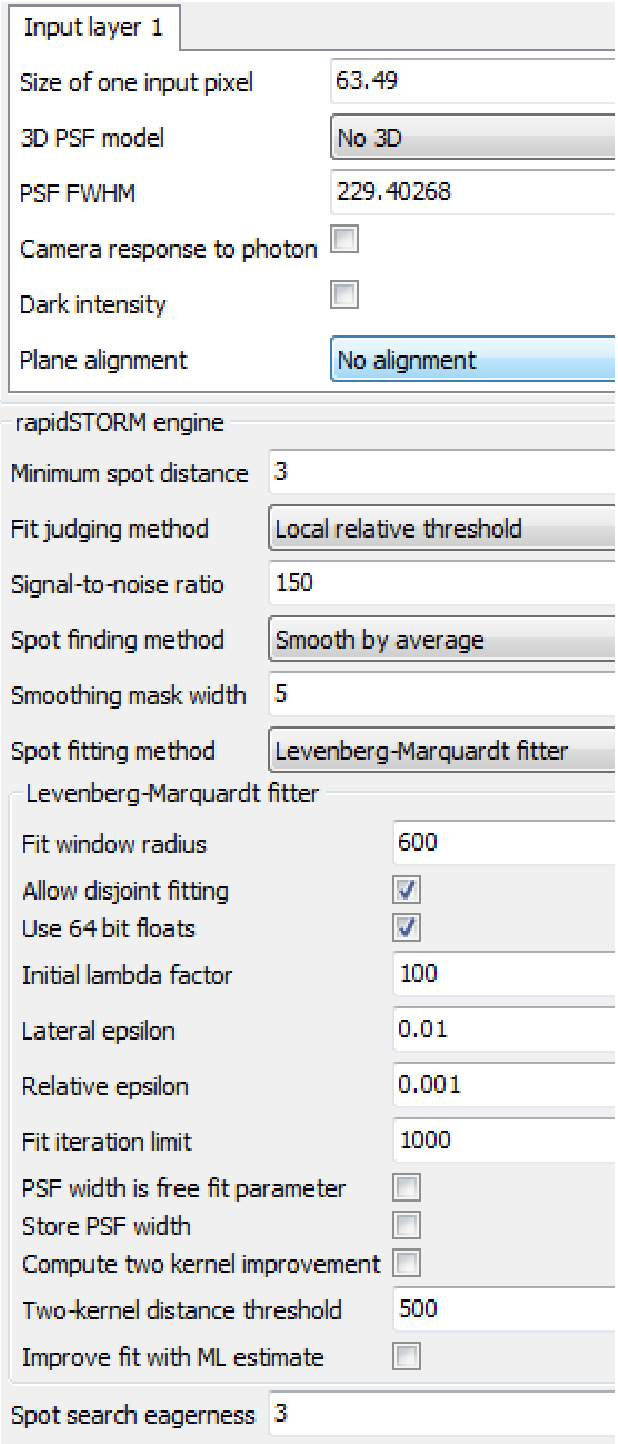

Figure S4: Parameters setting in rapidSTORM (high SNR)

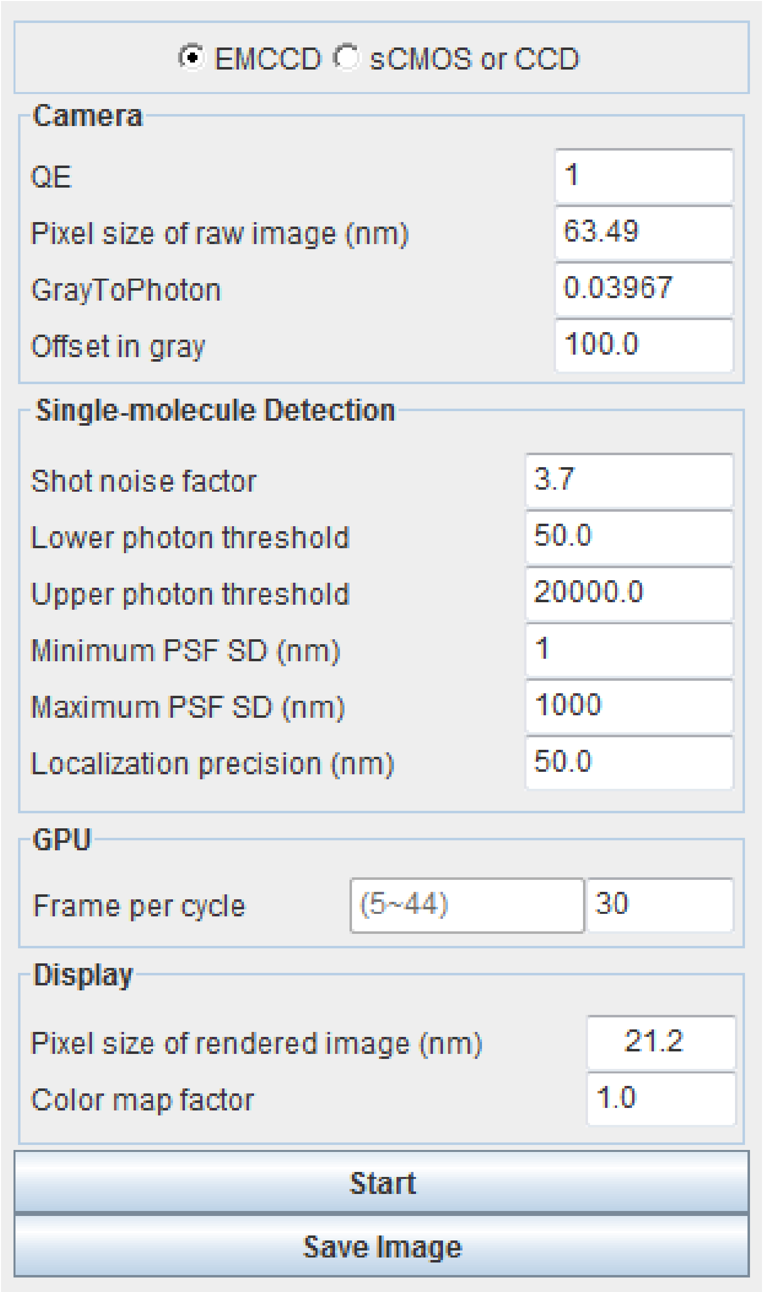

Figure S5: Parameters setting in MaLiang (high SNR)

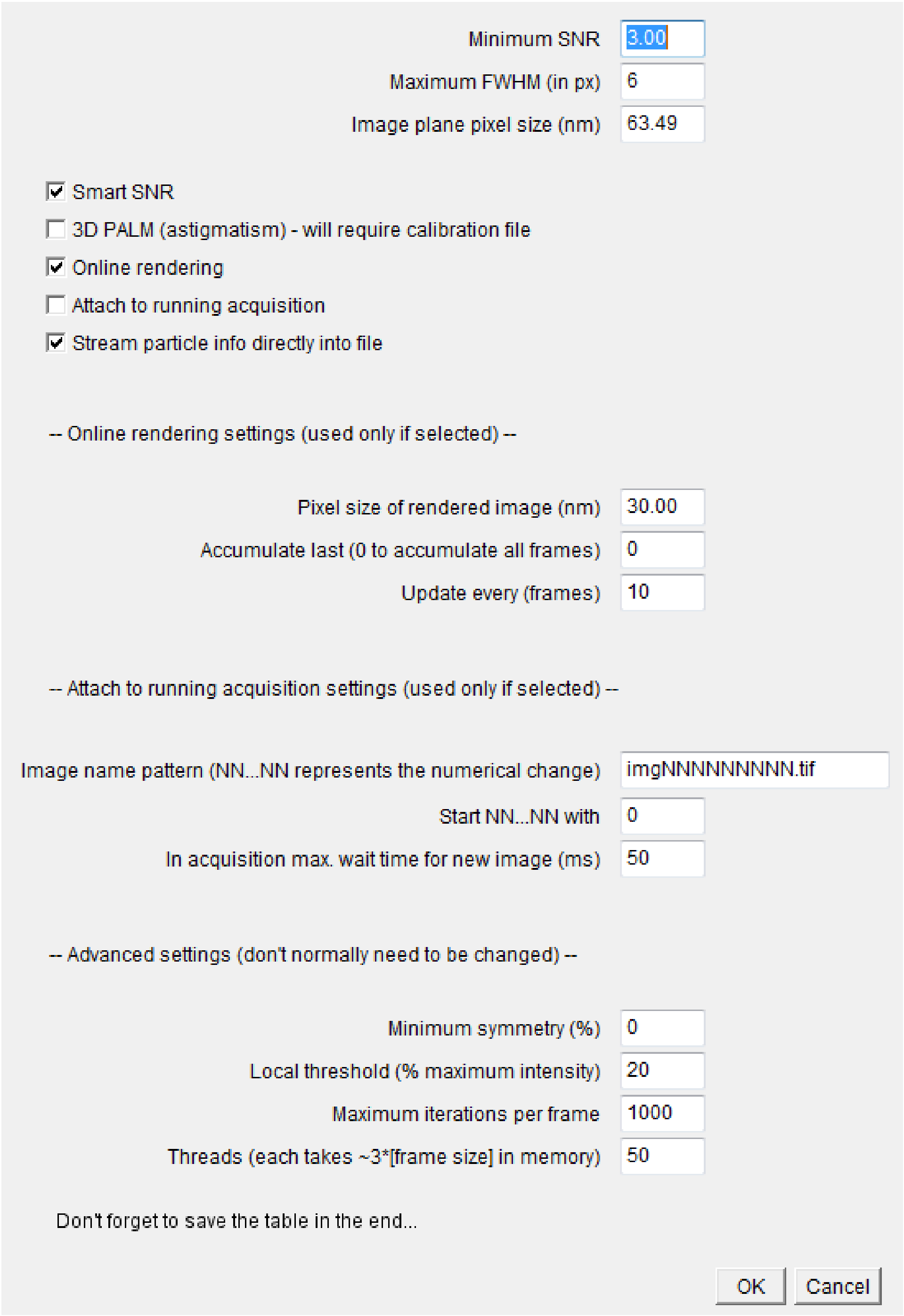

Figure S6: Parameters setting in QuickPALM (high SNR)

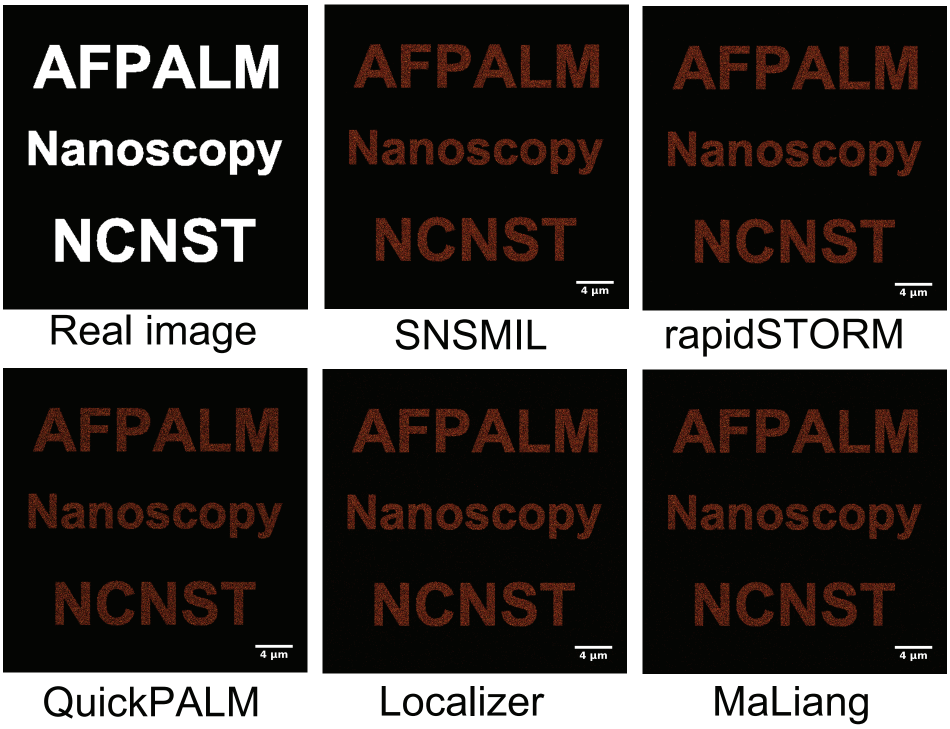

Figure S7: Reconstructed super-resolution images of high SNR simulated dataset.

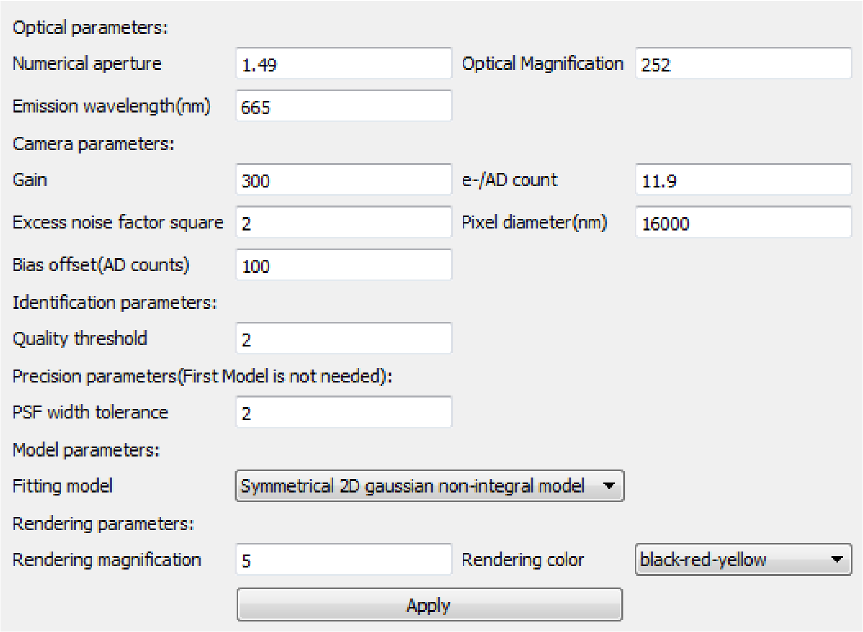

Figure S8: First parameters setting in SNSMIL (low SNR)

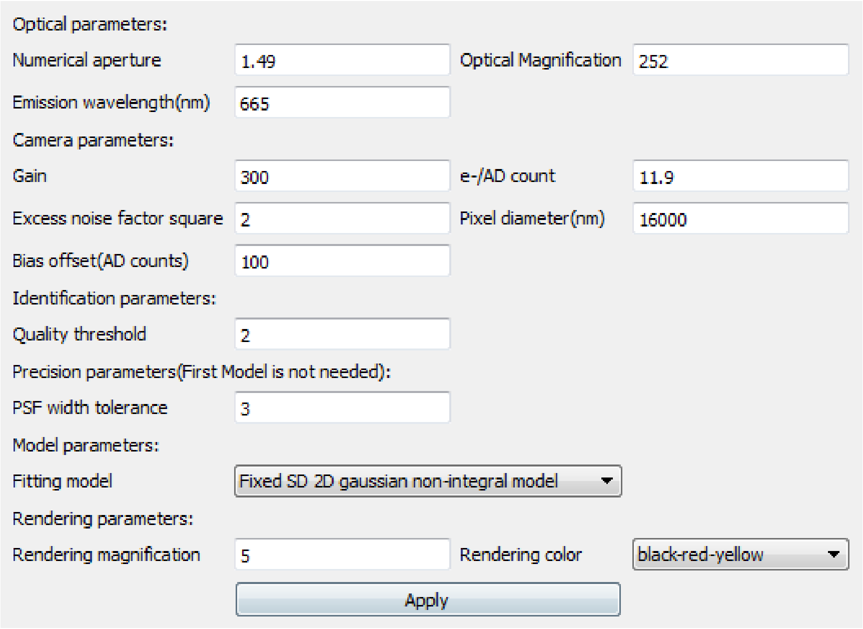

Figure S9: Second parameters setting in SNSMIL (low SNR)

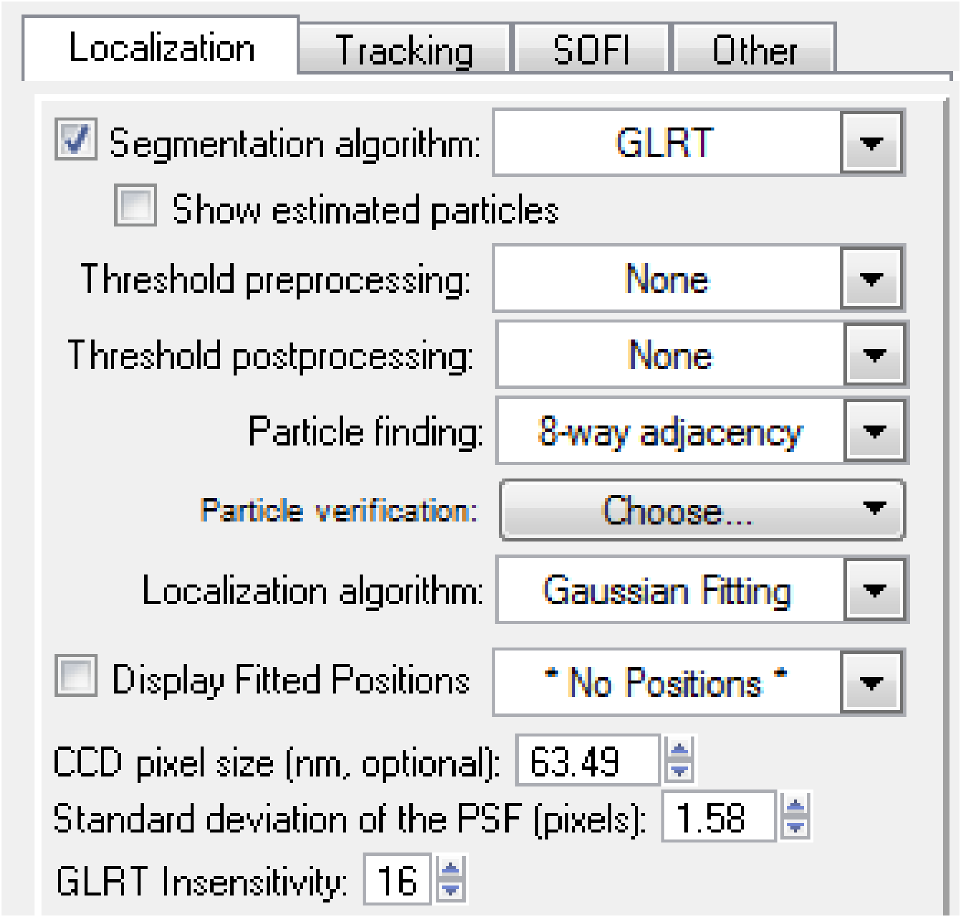

Figure S10: Parameters setting in Localizer (low SNR)

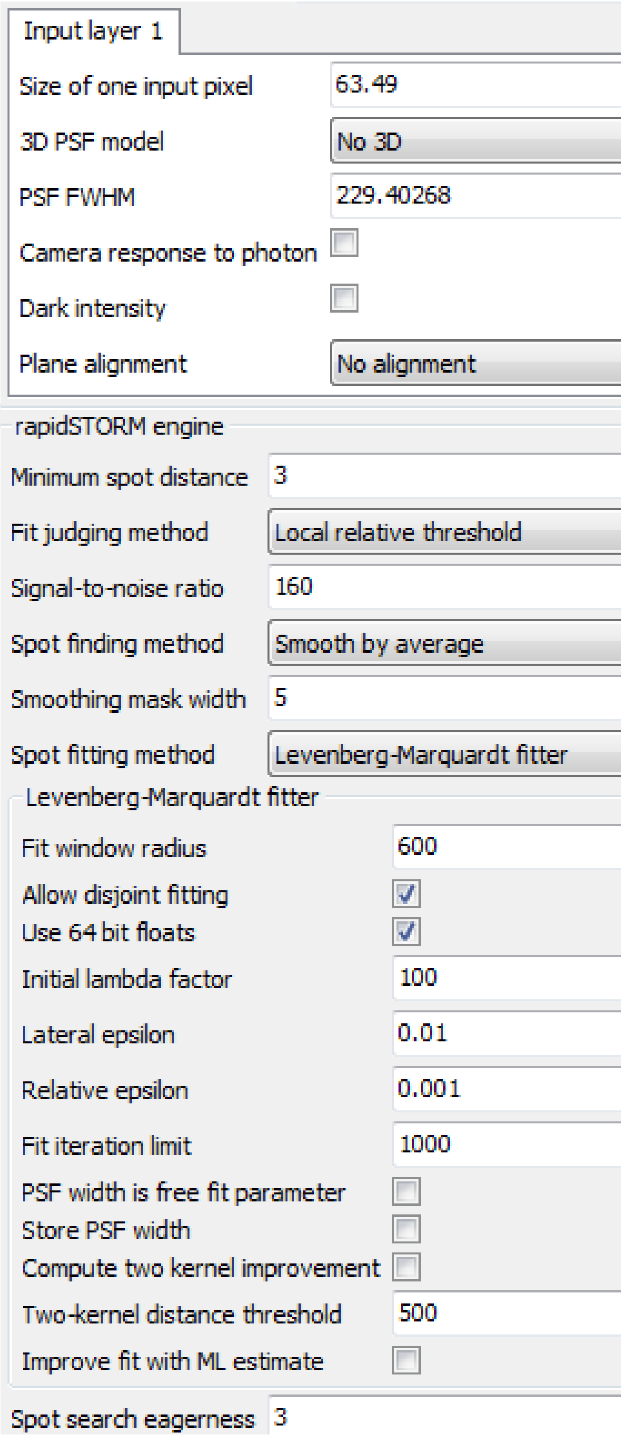

Figure S11: Parameters setting in rapidSTORM (low SNR)

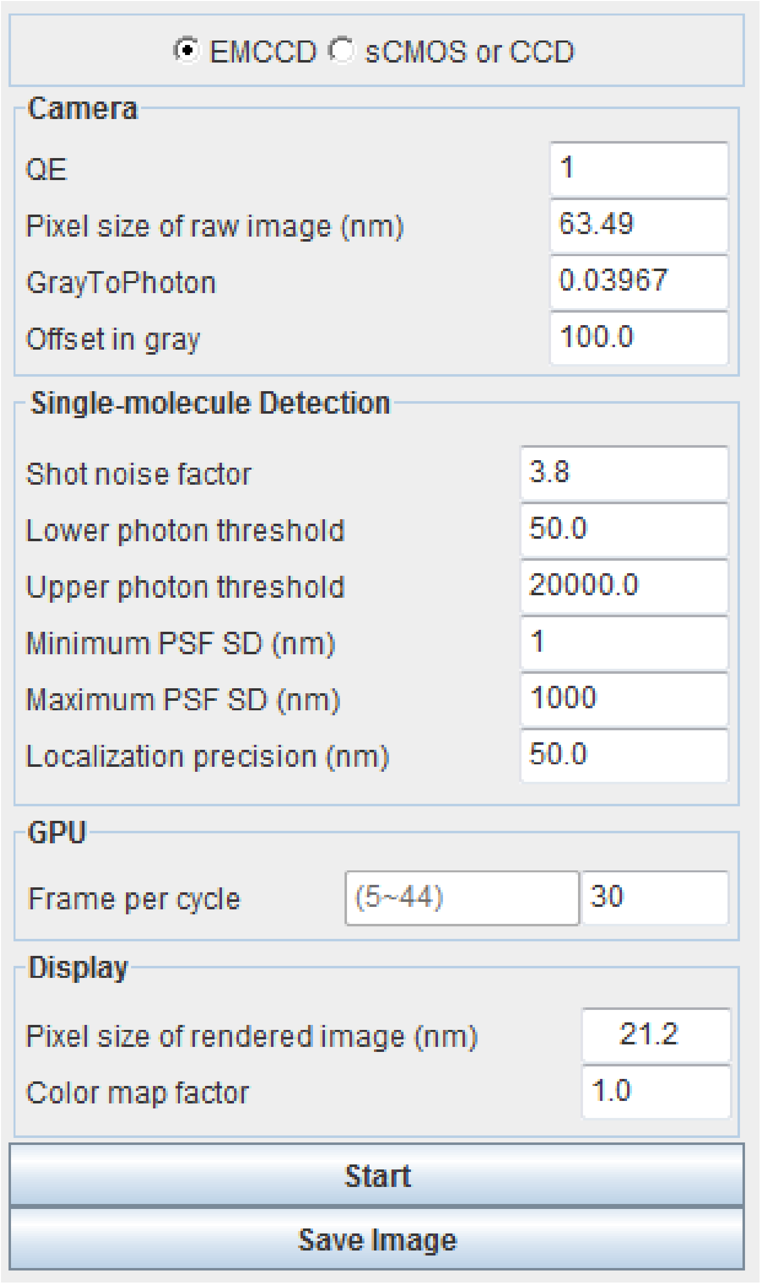

Figure S12: Parameters setting in MaLiang (low SNR)

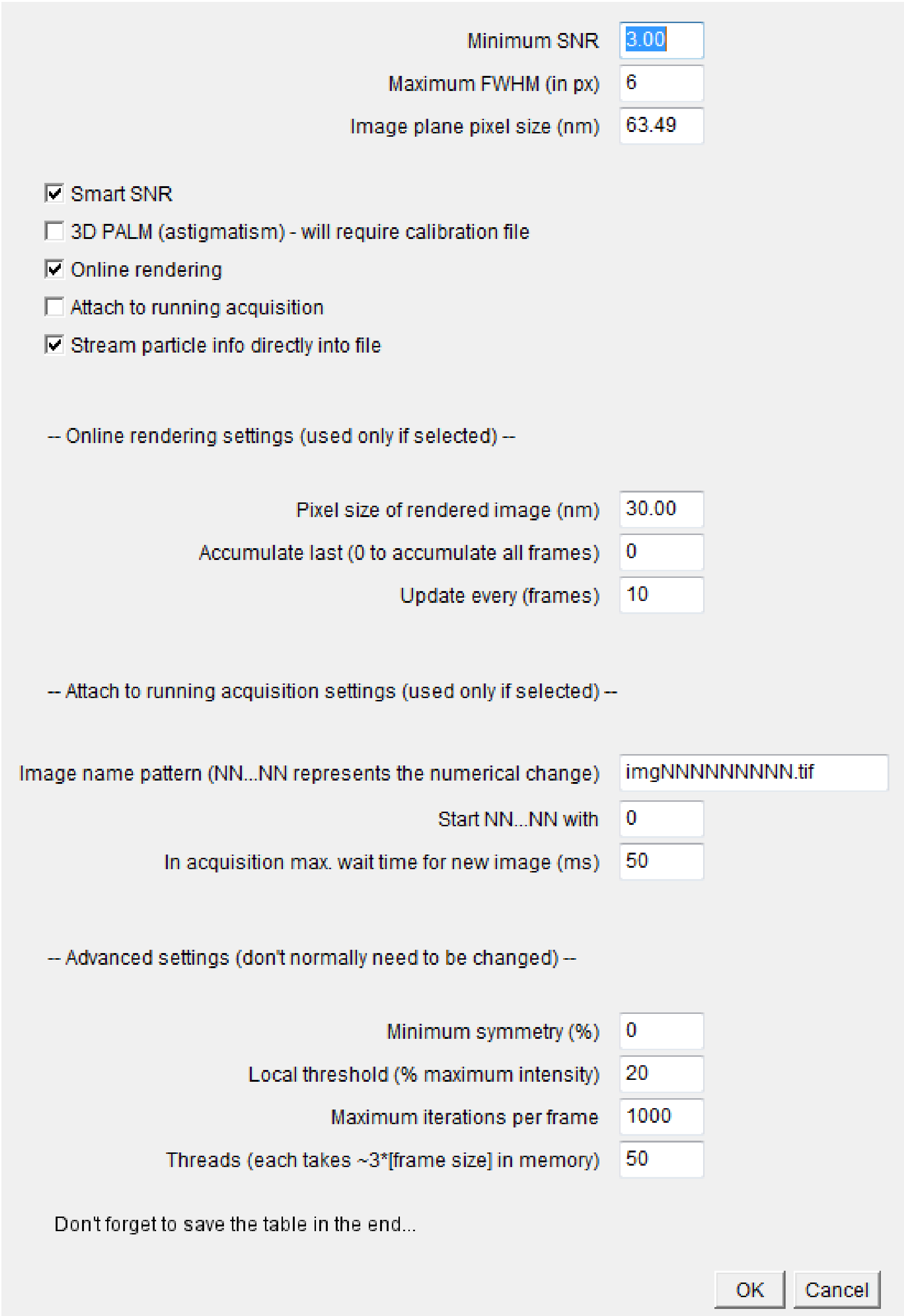

Figure S13: Parameters setting in QuickPALM (low SNR)

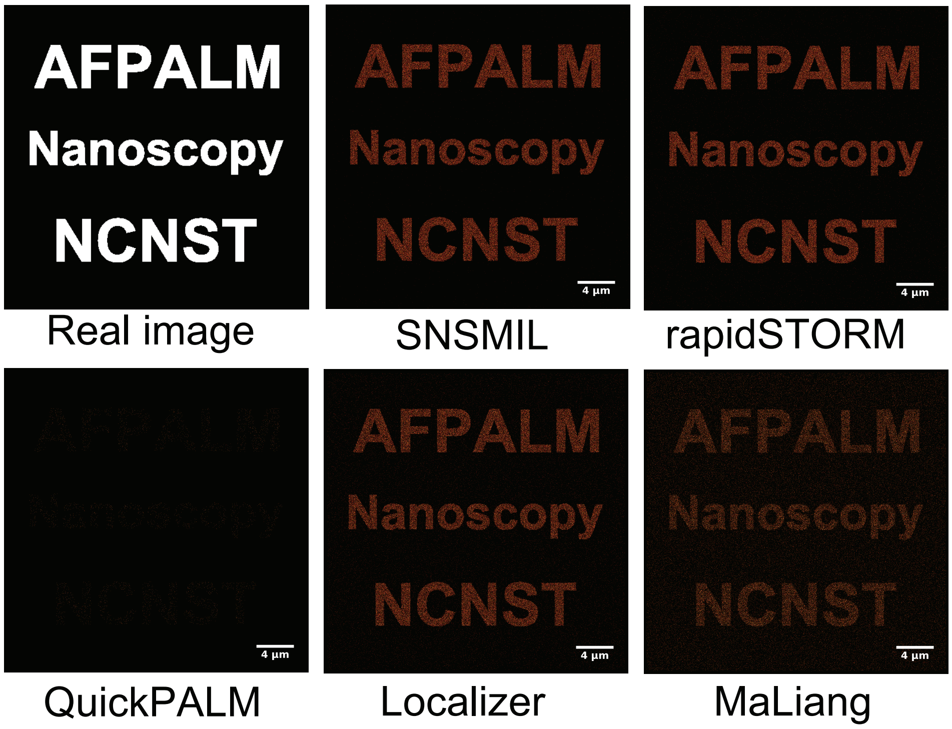

Figure S14: Reconstructed super-resolution images of low SNR simulated dataset.

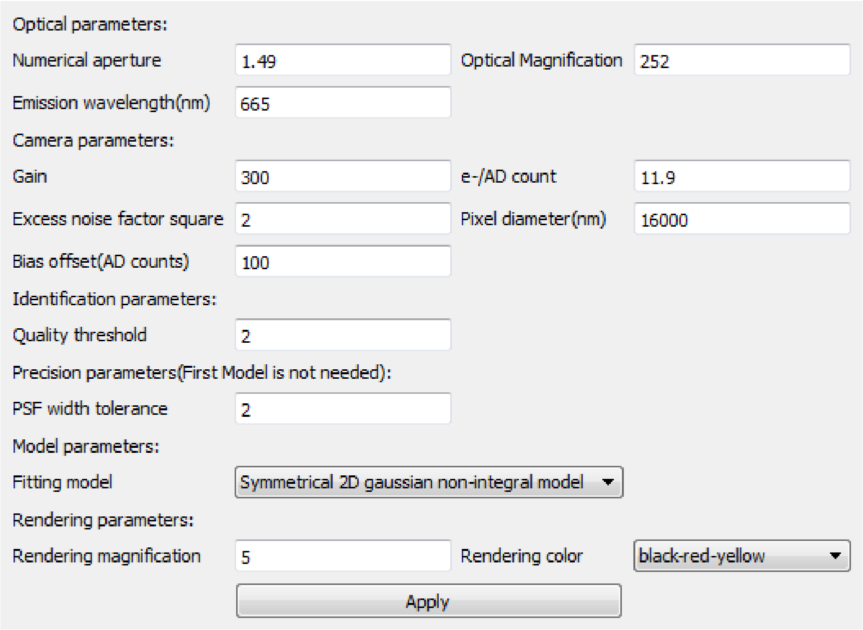

Figure S15: First parameters setting in SNSMIL (low SNR with GB)

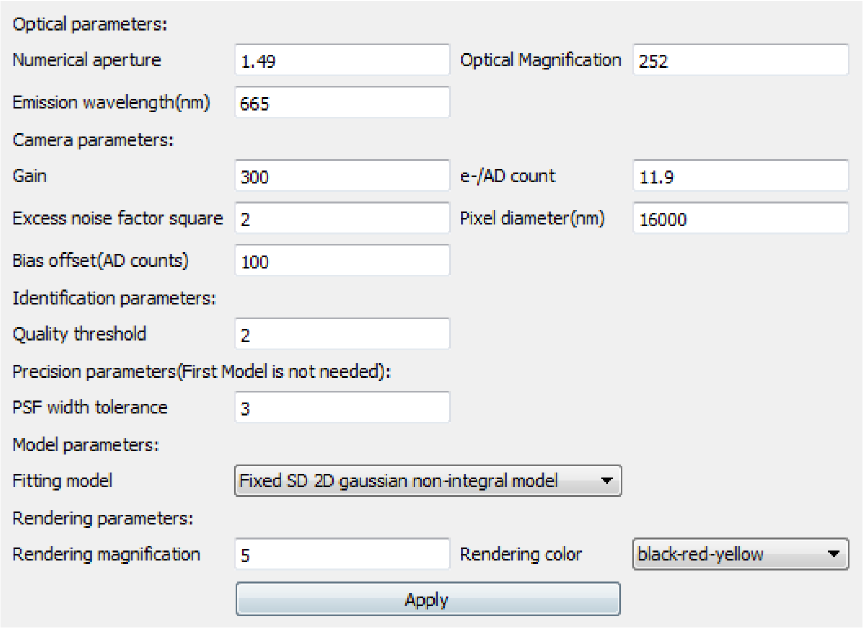

Figure S16: Second parameters setting in SNSMIL (low SNR with GB)

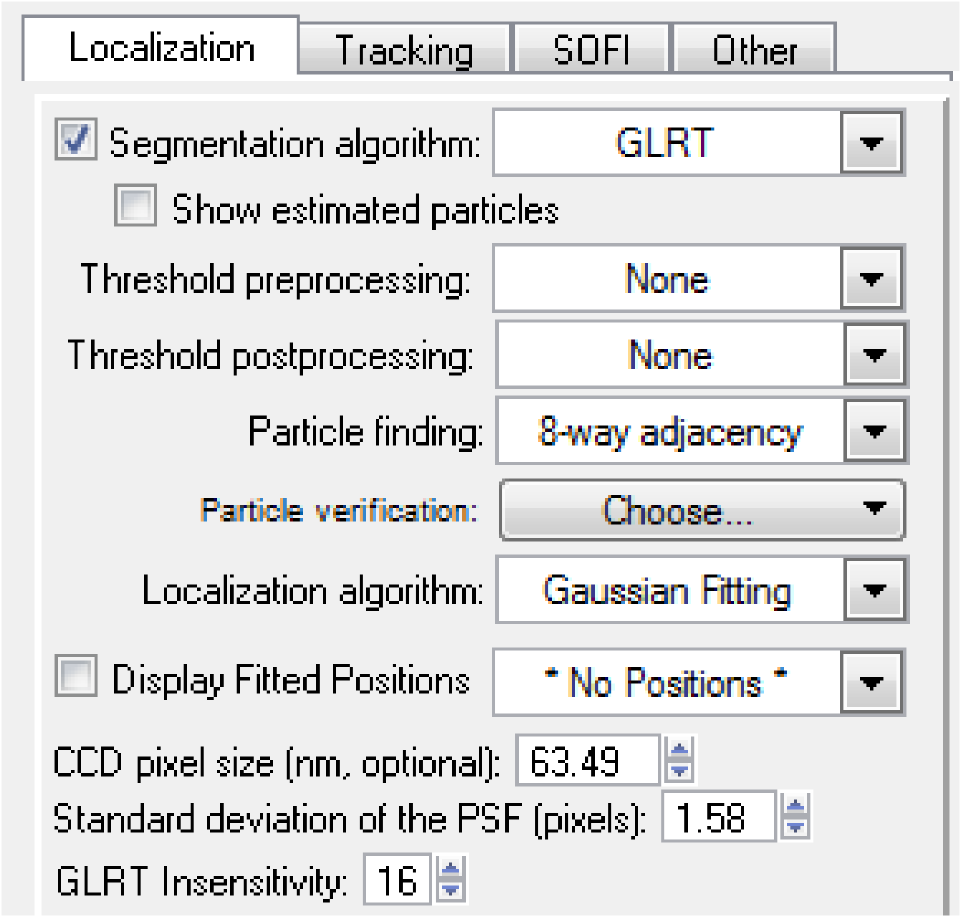

Figure S17: Parameters setting in Localizer (low SNR with GB)

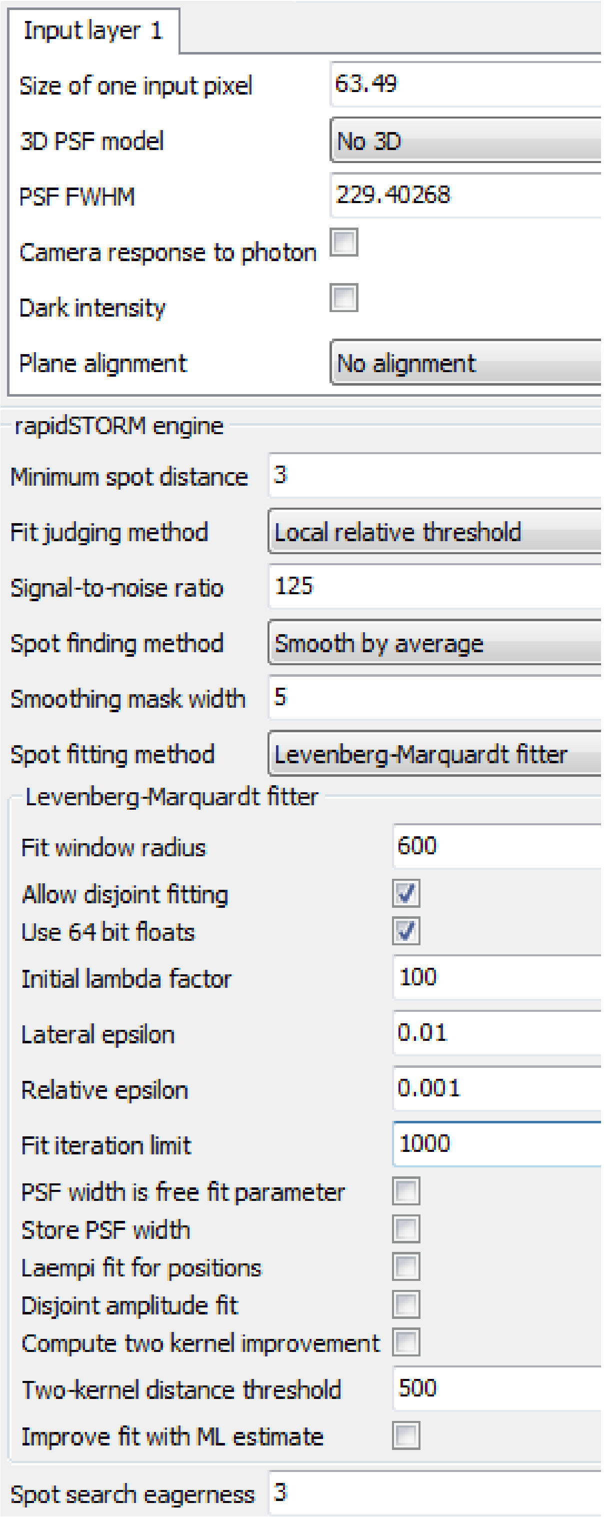

Figure S18: Parameters setting in rapidSTORM (low SNR with GB)

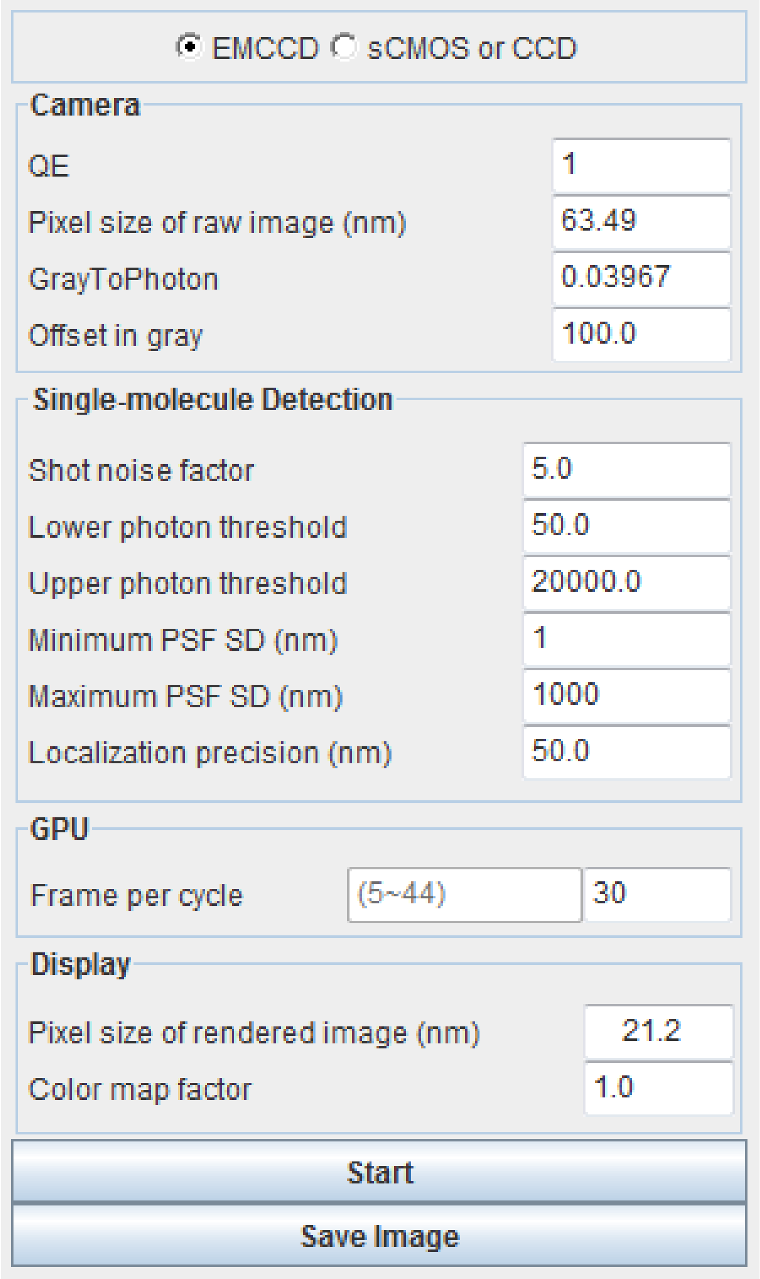

Figure S19: Parameters setting in MaLiang (low SNR with GB)

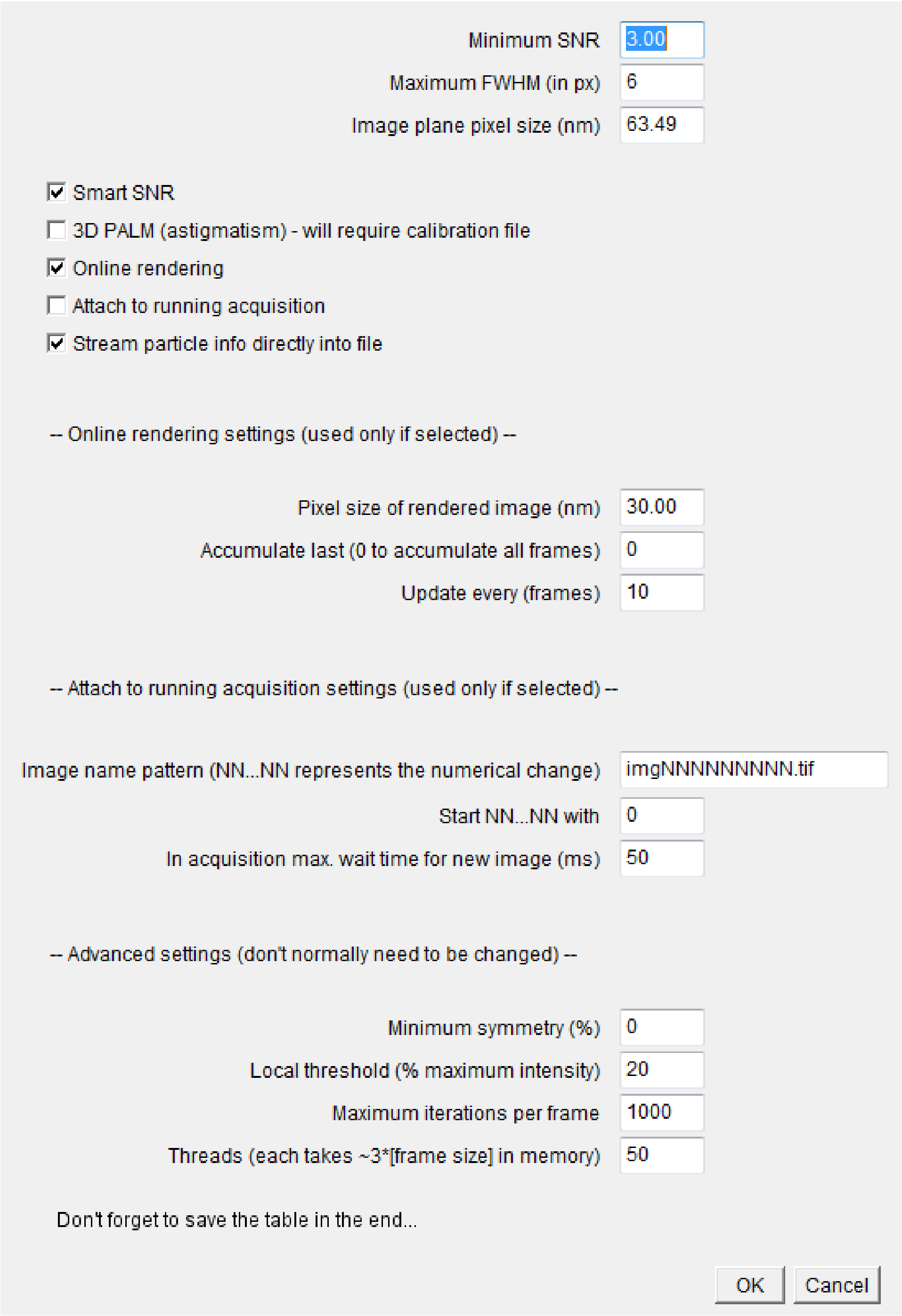

Figure S20: Parameters setting in QuickPALM (low SNR with GB)

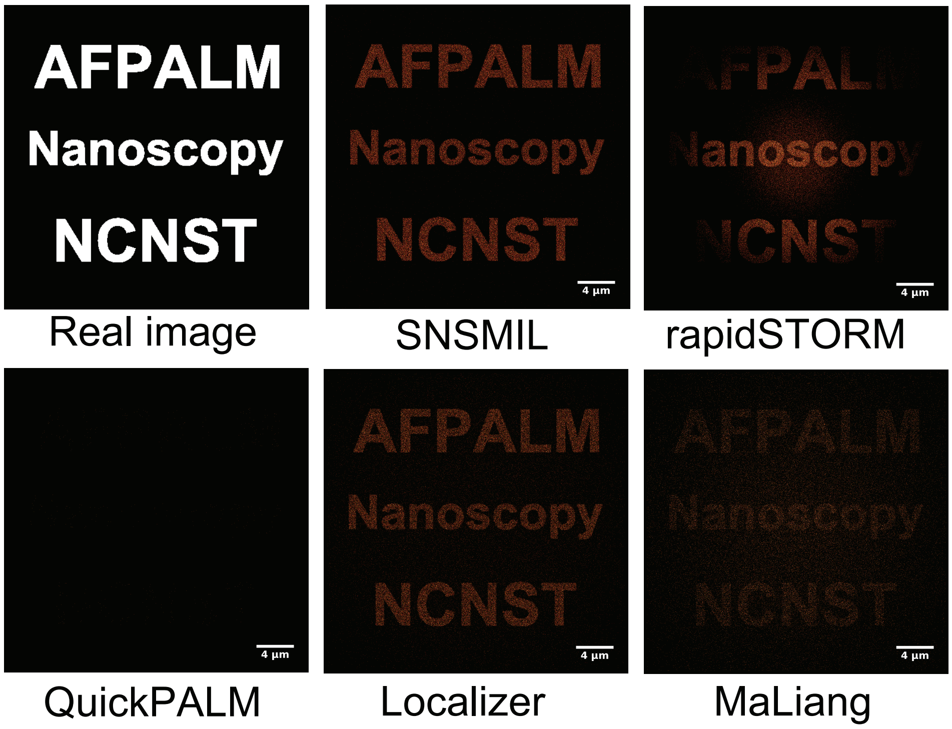

Figure S21: Reconstructed super-resolution images of low SNR with GB simulated dataset.

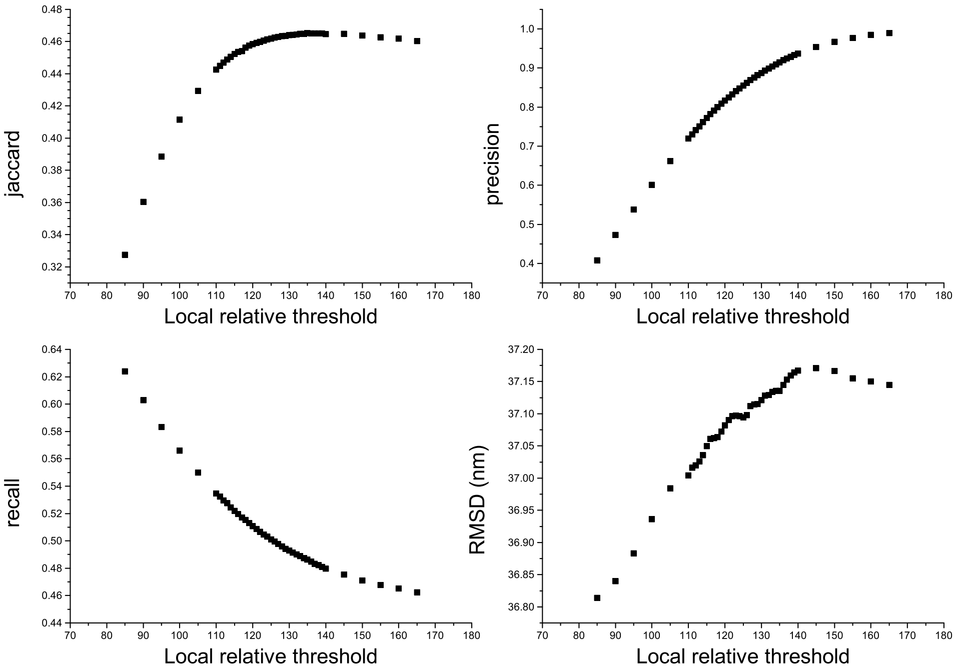

Figure S22: The different parameters setting of rapidSTORM for low SNR with GB simulated dataset.
